# Multireference and Coupled-Cluster Study of Dimethyltetroxide (MeO_4_Me) Formation and Decomposition

**DOI:** 10.1021/acs.jpca.3c08043

**Published:** 2024-02-28

**Authors:** Vili-Taneli Salo, Jing Chen, Nino Runeberg, Henrik G. Kjaergaard, Theo Kurtén

**Affiliations:** † Department of Chemistry, Faculty of Science, University of Helsinki, Helsinki FI-00014, Finland; ‡ Department of Chemistry, University of Copenhagen, Copenhagen 2100, Denmark

## Abstract

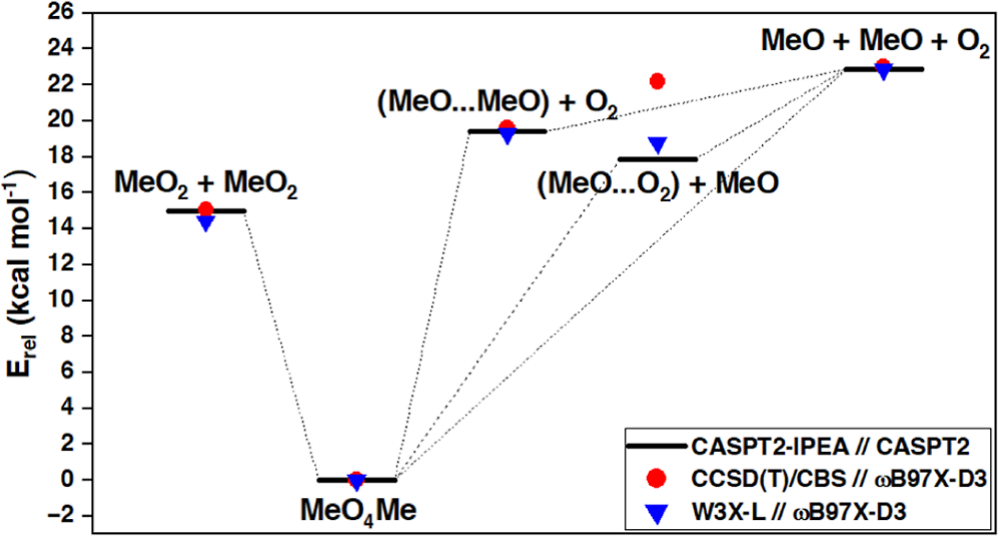

Peroxyl radicals (RO_2_) are important intermediates in the atmospheric oxidation processes. The RO_2_ can react with other RO_2_ to form reactive intermediates known as tetroxides, RO_4_R. The reaction mechanisms of RO_4_R formation and its various decomposition channels have been investigated in multiple computational studies, but previous approaches have not been able to provide a unified methodology that is able to connect all relevant reactions together. An apparent difficulty in modeling the RO_4_R formation and its decomposition is the involvement of open-shell singlet electronic states along the reaction pathway. Modeling such electronic states requires multireference (MR) methods, which we use in the present study to investigate in detail a model reaction of MeO_2_ + MeO_2_ → MeO_4_Me, and its decomposition, MeO_4_Me → MeO + O_2_ + MeO, as well as the two-body product complexes MeO···O_2_ + MeO and MeO···MeO + O_2_. The used MR methods are benchmarked against relative energies of MeO_2_ + MeO_2_, MeO_4_Me, and MeO + MeO + O_2_, calculated with CCSD(T)/CBS, W2X, and W3X-L methods. We found that the calculated relative energies of the overall MeO_2_ + MeO_2_ → MeO_4_Me → MeO + O_2_ + MeO reaction are very sensitive to the chosen MR method and that the CASPT2(22e,14o)-IPEA method is able to reproduce the relative energies obtained by the various coupled-cluster methods. Furthermore, CASPT2(22e,14o)-IPEA and W3X-L results show that the MeO···O_2_ product complex + MeO is lower in energy than the MeO···MeO complex + O_2_. The formation of MeO_4_Me is exothermic, and its decomposition is endothermic, relative to the tetroxide. Both the formation and decomposition reactions appear to follow pathways with no saddle points. According to potential energy surface scans of the decomposition pathway, the concerted cleavage of both MeO···O bonds in MeO_4_Me is energetically preferred over the corresponding sequential decomposition.

## 1 Introduction

**Scheme 1:**
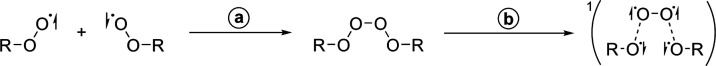
Tetroxide Formation from Two Peroxyl Radicals (a), and Its Decomposition to an Unstable Open-Shell Singlet RO···O_2_···RO Complex with Four Unpaired Electrons (b) See Scheme 2 for further product channels.

Peroxyl radicals (RO_2_) are reactive intermediates formed in atmospheric processes of volatile organic compounds.^1−6^ Bimolecular reactions are a major sink for RO_2_ under atmospheric conditions.^7,8^ In an urban atmosphere, the reactions with either NO or NO_2_ dominate the bimolecular reactions of RO_2_. Under pristine, low NO_*x*_ atmosphere, unimolecular reactions of RO_2_, and other bimolecular reactions, such as with HO_2_ or other RO_2_, may become competitive as well. The latter reaction is believed to proceed via the formation of an intermediate tetroxide (RO_4_R) species.^9−13^ Some of these tetroxides, such as the dimethyltetroxide (MeO_4_Me), have been observed in low-temperature matrix isolation experiments,^14^ while larger tetroxides have also been reported in low-temperature liquid-phase experiments.^12,15^ However, at ambient conditions, tetroxides have not been observed due to their exergonic decomposition in these conditions to form two alkoxyl radicals (RO) and molecular oxygen (O_2_) (Scheme 1). The decomposition occurs through an unstable RO···O_2_···RO complex^a^ (Scheme 1b) with four unpaired electrons.

**Scheme 2:**
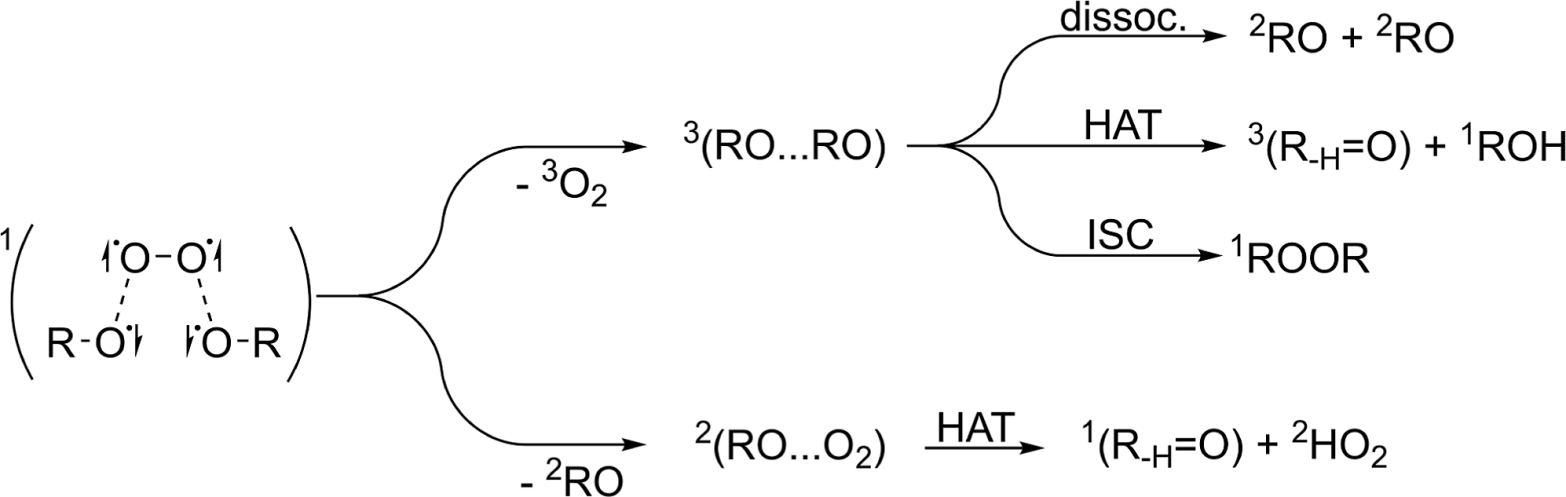
Potential Reaction Pathways Starting from the Unstable Open-Shell Singlet RO···O_2_···OR Complex Dissociation of molecular oxygen (top) or alkoxyl radical (bottom), followed by the corresponding product formation reactions. ISC = intersystem crossing.

Although the RO_4_R species is not observed under atmospheric conditions, the various decomposition products (Scheme 2) have been observed in the gas phase.^16,17^ Experiments show that the RO_2_ + RO_2_ reaction mainly branches into three product channels: (1) decomposition into two RO radicals and O_2_, (2) alcohol + carbonyl compound formation via hydrogen atom transfer (HAT) between the two RO radicals, and (3) peroxide (ROOR) formation from the addition of the two RO radicals.^18−20^ Furthermore, the RO and O_2_ may also react via HAT reaction to form a carbonyl compound and a hydroperoxyl radical (HO_2_).^21^ More complex RO_2_ may have yet more reaction channels available via in-complex RO + RO reactions.^22^

The branching of these product channels is likely strongly linked to the relative stabilities of the decomposition intermediates RO···RO + O_2_, RO···O_2_ + RO (and perhaps RO···O_2_···RO, while not necessarily for R = Me), which are not well understood currently. Understanding how various functional groups in RO affect the ROOR formation is central to the studies of organic new-particle formation in the atmosphere.

Due to the difficulty in the experimental detection of tetroxides, computational studies of its reaction pathways are of significant interest. The reaction has been studied with various computational approaches, such as ab initio methods^23−26^ and trajectory-based methods.^27,28^ The standard single-reference (SR) electronic structure methods, such as density functional theory (DFT) or coupled cluster (CC), can be used to obtain accurate relative energies for separate, noninteracting species (RO, RO_2_, and O_2_), as well as for the RO_4_R. The ground electronic states of these molecules can be qualitatively described with a single-determinant approximation, i.e., with the Hartree–Fock (HF) method.

For the reaction pathways connecting these species, the single-determinant approximation does not hold, since the formation reaction, RO_2_ + RO_2_ → RO_4_R, occurs on an open-shell singlet state (Scheme 1a).^29^ In the decomposition reaction (RO_4_R → RO + O_2_ + RO), the molecular oxygen is likely formed in its triplet ground state, as the lowest singlet state is more than 20 kcal mol^–1^ above the triplet state in energy.^30^ Therefore, the decomposing tetroxide likely yields two doublet RO and a triplet O_2_, all coupled as an overall open-shell singlet with four unpaired electrons (Scheme 1b). An accurate description of open-shell singlet states requires the use of multireference methods (MR),^31,32^ which appropriately consider the strong correlation effects in such electronic structures. These MR methods are expensive and nontrivial to use properly.

To overcome the difficulties in handling open-shell singlet states, many previous studies have so far assumed that the O_2_ is only weakly bound after the decomposition and irrelevant for subsequent reactions, thus being excluded from further calculations. Within this approximation, the product channels starting from the triplet complex of RO···RO (top pathway in Scheme 2) have been studied using various SR methods.^33−35^

Attempts have also been made to describe the reaction pathways using MR methods. However, the results are far from satisfactory.^25,26^ Previously, the CASSCF method was used for obtaining stationary structures along the reaction pathway and then the CASSCF surfaces were corrected with the extended multiconfigurational quasi-degenerate perturbation theory (XMC-QDPT2)^36^ dynamic correlation method.^26^ The performance of the used methodology was erratic, and it also did not yield convincing evidence for the reaction mechanism, especially regarding the decomposition reaction. Furthermore, none of the calculated overall reaction energies for the RO_2_ + RO_2_ → RO_4_R → RO + RO + O_2_ reaction agreed with energies calculated with coupler-cluster single-point energy corrections on DFT optimized geometries (CC//DFT). The coupled-cluster method can be used when HF converges to a qualitatively correct state, which applies to all components of the overall reaction, as long as they are not interacting with each other (including the covalently bound tetroxide). If a multireference approach cannot reproduce the CC relative energies of the isolated reaction components with sufficient accuracy, then there is little confidence that such an approach would produce credible results on other parts of the reaction potential energy surfaces.

In this study, we further explore the various variables and settings of multireference methods in the context of the RO_2_ + RO_2_ → RO_4_R → RO + RO + O_2_ reactions. We begin by generating high accuracy relative energy estimates with composite coupled-cluster methods^37^ for O_2_, RO, RO_2_, and RO_4_R, to provide the best possible CC description for benchmarking purposes. Then, we identify an appropriate multireference methodology that accurately reproduces the CC//DFT energy profile accurately. Due to the high cost of the methods, only MeOOOOMe (R = Me) is used in the present study of the RO_2_ + RO_2_ → RO_4_R → RO + RO + O_2_ reactions. Additionally, we show that for the model system, the assumption that O_2_ is weakly bound does not hold, instead, the triplet MeO···MeO complex is less bound than the MeO···O_2_ complex. We build a foundation for a more rigorous investigation of the postdecomposition reactions that require using multireference methods. We intend to study such reactions along with other RO_2_ systems in future studies.

## 2 Computational Methods

### 2.1 Density Functional Theory Methods

We used DFT methods for obtaining structures for various coupled-cluster calculations as well as initial structures for multireference geometry optimizations. We used ωB97X-D3^38^ and the M06-2X functionals,^39^ as implemented in ORCA version 5.0.3.^40,41^ With M06-2X, an empirical dispersion correction with zero-damping function (D3Zero) was used.^42^ The ωB97X-D3 functional already includes a dispersion correction, which is also based on the zero-damping scheme. Both of these functionals are hybrid functionals. M06-2X has a fixed, 54% HF exchange, and ωB97X-D3 has a range-separated HF exchange, i.e., the amount of HF exchange depends on interelectronic distances.

All the DFT geometry optimizations and frequency calculations were done with the fully augmented, triple-ζ correlation-consistent basis set, aug-cc-pVTZ.^43,44^ We used strict convergence criteria for both the energies and geometries. Furthermore, M06-2X calculations were performed using tighter DFT integration grids (DefGrid3 instead of the default DefGrid2 in ORCA-5.0.3). Frequency calculations were carried out to ensure that the optimized geometries corresponded to true minimum energy stationary points.

### 2.2 Coupled-Cluster Methods

Coupled-cluster (CC) calculations were carried out to obtain the accurate energies. We used the coupled-cluster singles and doubles with perturbative triples (CCSD(T)) method with complete basis set (CBS) limit extrapolation. The CC calculations were done using cc-pVDZ, cc-pVTZ, and cc-pVQZ basis sets,^43^ and the CBS extrapolations were calculated using the automatic CBS-extrapolation in ORCA. The SCF part of the extrapolation is calculated with the following scheme^45,46^
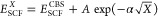
and the correlation part is calculated with^47^
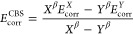
in which *X* and *Y* are successive cardinal numbers in the basis set series. The constant *A* is determined during the extrapolation, and α and β are basis set and extrapolation scheme-specific constants. Two-point extrapolations using either cc-pVDZ and cc-pVTZ or cc-pVTZ and cc-pVQZ were used to obtain CBS energies, denoted hereinafter as CBS(2/3) or CBS(3/4). Unless stated otherwise, the CCSD(T)/CBS energies correspond to the CBS(3/4) extrapolation. Coupled-cluster calculations were done using ORCA, Molpro,^48−50^ and MRCC^51,52^ softwares.

In addition to canonical CC calculations, we also did benchmarks using the W2X and W3X-L composite methods,^37^ which are the most computationally demanding composite methods feasible in our computational facilities. The W2X procedure provides an accurate approximation of the all-electron scalar-relativistic CCSD(T)/CBS energy. The W3X-L protocol incorporates also post-CCSD(T) effects up to CCSDT(Q), which are crucial in predicting the accurate reaction energetics for reactions that involve strong electron correlation.^53^

All of the components required to construct the W2X energies were obtained using converged Restricted Hartree–Fock (RHF) orbitals and the restricted versions of the correlated methods, as implemented in Molpro. The final W3X-L energies were assembled by adding corrections from CCSDT and CCSDT(Q) calculations that were performed using MRCC.

### 2.3 Complete Active Space Setup

We constructed three complete active spaces (CAS) for studying the tetroxide reactions. The smallest of these active spaces, (6e,6o) was constructed from the three σ and σ* orbitals of the MeO–O–O–OMe structure. This is the smallest active space that can potentially qualitatively describe the formation of a tetroxide from two peroxyl radicals as well as its decomposition products, molecular oxygen, and two alkoxyl radicals. The next active space, (10e,8o), contains all orbitals of the (6e,6o) set and two additional lonepair/π orbitals localized in the inner O–O oxygen atoms. These orbitals correspond to the occupied π orbitals of the atom of O_2_ in the decomposition. The largest active space, CAS(22e,14o), incorporates all oxygen valence orbitals, except C–O σ and σ* orbitals. The various optimized active space orbitals are shown in the Supporting Information (Figures S1–S3). The CAS wave functions were optimized using the complete active space self-consistent field method (CASSCF). We used the perturbation-based orbital update (SuperCI_PT) scheme,^54^ with very tight criteria for energy (1 × 10^–10^ au) and orbital gradient (1 × 10^–6^ au) convergence in the CASSCF wave function optimization.

### 2.4 Multireference Perturbation Theory Methods

We used various multireference perturbation theory (MRPT) methods for treating the dynamical correlation in the studied systems. The used MRPT methods are either based on complete active space second-order perturbation theory (CASPT2) or N-electron valence state second-order perturbation theory (NEVPT2).^55−58^ The main difference between these methods is in the definition of the zeroth-order Hamiltonian for the CAS reference wave function. CASPT2 uses a generalized Fock operator that in the closed-shell limit yields energies identical to MP2,^59^ while NEVPT2 uses a partially bielectronic Dyall Hamiltonian.^60^ The Dyall Hamiltonian treats the inactive occupied and virtual orbital space with a one-electron Fock-operator and the CAS with a bielectronic Hamiltonian.

All CASPT2 and NEVPT2 calculations in this work employed the fully internally contracted variant of the theory (FIC-CASPT2 and FIC-NEVPT2, in early literature also called partially contracted).^57,61^ Due to the use of large active spaces (22e,14o), the evaluation of third and fourth-order density matrices in NEVPT2 turned out to be a computational bottleneck.^58^ Thus, the newer efficient variant of NEVPT2 was used, to avoid the explicit evaluations of higher-order reduced density matrices.^62^ Additionally, strict prescreening criteria for the 3-RDM and 4-RDM (1 × 10^–16^ au) were used to eliminate any false intruder states arising from approximated density matrices.^63^

CASPT2 has been shown to underestimate dissociation energies for systems, where the number of paired electrons change.^64,65^ The dissociation energies are underestimated by 2–5 kcal mol^–1^ for each changed electron pair.^65^ The error is due to the use of the generalized Fock-operator, which depends on the one-electron Fock matrices. The diagonal elements of these matrices (orbital energies) can be expressed in terms of the negative ionization potential (IP) for occupied orbitals, and in terms of the negative electron affinity (EA) for unoccupied orbitals, as per the extended Koopmans’ theorem.^66−68^ This allows interpreting the changes in orbital energies in PT2 substitutions in terms of IP and EA. The use of Koopmans’ theorem for assigning energies for orbitals in the PT2 substitutions is only justified when the participating orbitals have integer occupations of 2.0 or 0.0; i.e., the orbitals are eigenfunctions of the restricted Fock operator. Because CAS orbitals or unrestricted open-shell orbitals are not eigenfunctions of the restricted Fock operator, their orbital energies do not carry a physical interpretation similar to that of RHF orbitals. It has been shown that using open-shell orbital or CAS orbital energies in the PT2 energy corrections yields too small denominators in the perturbation expression, which in turn leads to overestimated correlation energies in such systems.

In addition to CASPT2, we used IPEA-shifted CASPT2 (CASPT2-IPEA).^65^ In CASPT2-IPEA, when the substitutions involve CAS-orbitals, the energy difference between IP and EA is not evaluated explicitly, but substituted with a parameter, which is referred to as IPEA-shift in literature.^65,69^ In this work, we benchmarked the value of IPEA-shift (see Supporting Information Section S4) and found that the value of 0.20 au works well for the purpose of this study. Additionally, we did thorough PT2 diagnostics with CASPT2 data to verify the applicability of perturbation theory to the studied chemical problems (Supporting Information Section S3).

## 3 Results and Discussion

We first show the CC/DFT benchmarking results. CCSD(T) and W3X-L methods are used to calculate accurate energies for each relevant species in the tetroxide formation and its decomposition reaction (MeO_4_Me, MeO_2_, MeO, ^3^O_2_). Additionally, the CC//DFT methods are used to calculate energies for the two-body complexes MeO···MeO and MeO···O_2_. The W3X-L and W2X calculations are done with spin pure R(O)HF references, while CCSD(T)/CBS calculations were done with spin unrestricted UHF reference, where the MeO···MeO is calculated at a triplet state and the MeO···O_2_ complex is a mixture of doublet and quartet states . The multireference results for these complexes also include the dissociated MeO/O_2_, so the total spin is a singlet. Then, we show the formation reaction pathway, MeO_2_ + MeO_2_ → MeO_4_Me, and the dissociation pathway, MeO_4_Me → MeO + MeO + O_2_, calculated using various multireference approaches. We demonstrate in detail, how various active spaces, dynamical correlation methods, and basis sets affect the overall energetics. Then, we use the appropriate MR approach to calculate relative energies for the MeO···MeO, and MeO···O_2_ stationary points as well. Finally, we discuss how the used multireference approach can benefit future research, also assessing its limitations.

### 3.1 W3X-L and CCSD(T)/CBS Benchmarks for Total Reaction Energies

We optimized the geometries of MeO_2_, MeO, O_2_, and MeO_4_Me, and the following two complexes: MeO···MeO and MeO···O_2_ at ωB97X-D3/aug-cc-pVTZ and M06-2X/aug-cc-pVTZ level to see how sensitive the CC relative energies are to geometries optimized with different DFT-functionals. The effect of these functionals on optimized geometries is discussed in the Supporting Information Section S6. Then, we calculated CCSD(T)/CBS single-point energy corrections using both sets of geometries. We found that the CCSD(T)/CBS relative energies are very similar to both used DFT-functionals (M06-2X/aug-cc-pVTZ results in Supporting Information Section S7). The W2X and W3X-L single-point energies were calculated using ωB97X-D3/aug-cc-pVTZ optimized geometries. Results of the CC//ωB97X-D3/aug-cc-pVTZ benchmark are shown in Table 1.

**Table 1:** CC//ωB97X-D3/aug-cc-pVTZ Relative Electronic Energies of MeO_2_ + MeO_2_, MeO_4_Me, MeO···MeO + O_2_, MeO···O_2_ + MeO, and MeO + MeO + ^3^O_2_, in kcal mol^–1^

method^a^	MeO_2_ + MeO_2_	MeO_4_Me	MeO···MeO^b^	MeO···O_2_^b^	MeO + MeO + ^3^O_2_
CC/cc-pVTZ	14.56	0.00	16.75	19.55	20.48
CC/cc-pVQZ	14.64	0.00	18.16	20.77	21.58
CC/CBS(2/3)	16.39	0.00	21.80	24.39	25.28
CC/CBS(3/4)	15.05	0.00	19.63	22.20	23.01
W2X	15.22	0.00	19.78	24.26	23.09
W3X-L	14.42	0.00	19.30	18.80	22.87

a CC: CCSD(T).

b MeO···MeO + ^3^O_2_ and MeO···O_2_ + ^2^MeO.

The benchmark results show that the energies of two MeO_2_ radicals are 14–15 kcal mol^–1^ above the MeO_4_Me tetroxide. Similarly, the decomposition products, two MeO radicals, and triplet O_2_ are 23 kcal mol^–1^ above the tetroxide in energy. The CCSD(T)/CBS, W2X, and W3X-L appear to agree on the relative energies on all stationary points except for the MeO···O_2_ complex. The 5.5 kcal mol^–1^ energy difference between W2X and W3X-L suggests that the MeO···O_2_ has a substantial multireference character. It has been shown for the analogous HO···O_2_ system that single-reference methods are not able to describe its structure properly.^70,71^

Although the CC methods cannot be used to calculate the reaction pathways, these relative energies are a good benchmark for the multireference methods discussed in the next section.

### 3.2 Formation Reaction, MeO_2_ + MeO_2_ → MeO_4_Me

In this section, we show how various parameters in multireference calculations affect the formation reaction profile. First, we investigate the effect of using different active spaces on the reaction potential energy surfaces optimized at the CASSCF level. Afterward, we treated dynamical correlation by adding CASPT2 and CASPT2-IPEA single-point energy corrections on the CASSCF-optimized surfaces. Then, we check how sensitive the relative energies are to the chosen basis set. Lastly, we optimize the geometries in the formation reaction profile also at the CASPT2 level to see whether an accurate description of dynamic correlation is necessary already in geometry optimizations.

**Figure 1:**
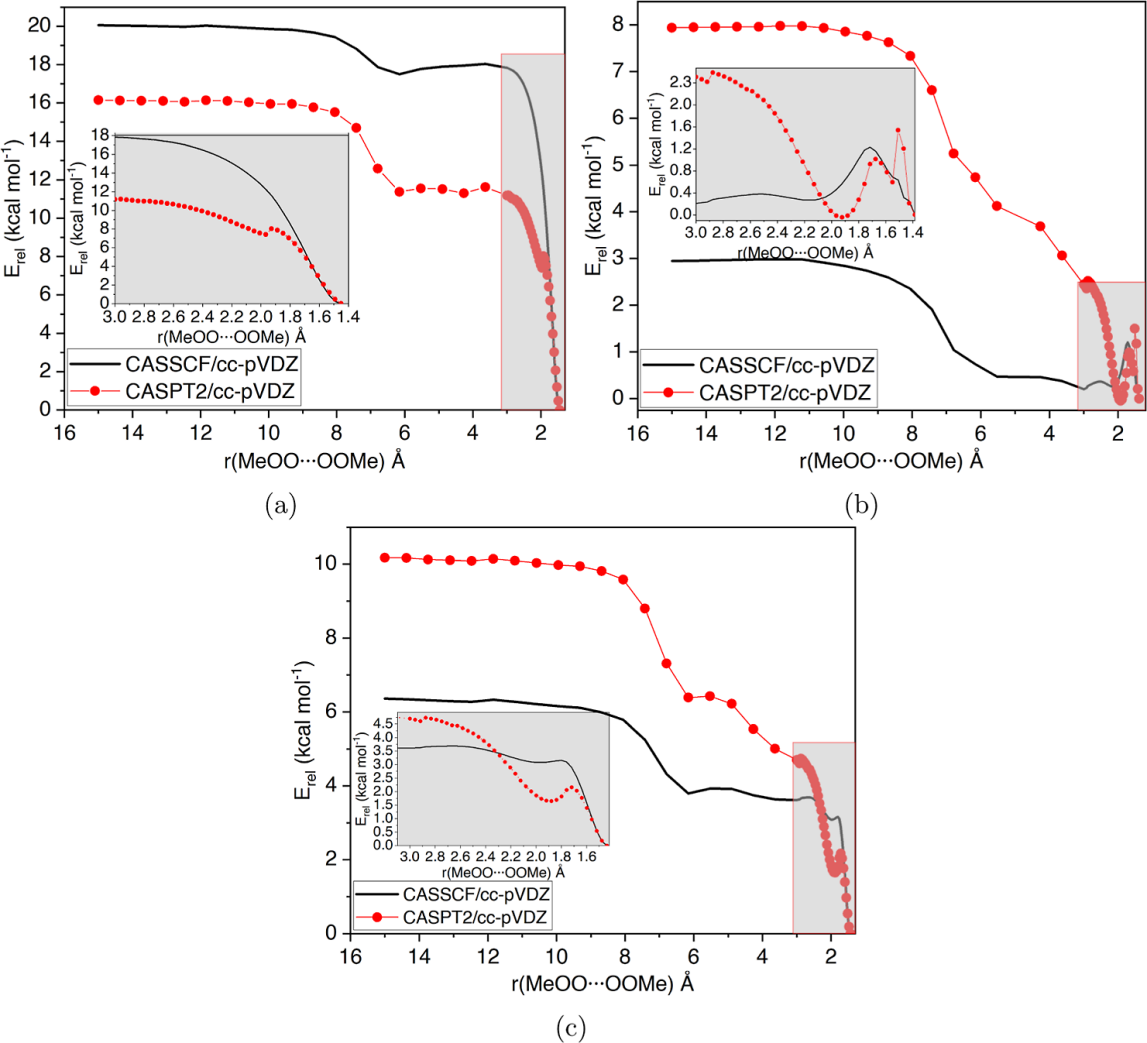
Relaxed scan of the MeOO···OOMe bond length. PES optimized with CASSCF, using (a) 6e,6o, (b) 10e,8o, and (c) 22e,14o active spaces. Circles correspond to CASPT2 single-point energy corrections.

We used three active spaces, (6e,6o), (10e,8o), and (22e,14o), to study the static electron correlation in the formation reaction (details of the active spaces in the Computational Methods section). With these three active spaces, we scanned the formation reaction potential energy curve (PES) by increasing the MeOO···OOMe distance up to 15 Å, starting from the tetroxide structure, at CASSCF/cc-pVDZ level of theory (Figure 1). During the scan, all other degrees of freedom, except the MeOO···OOMe distance, were relaxed. All PESs are calculated on a singlet state, where the doublet RO_2_ radicals are coupled as an open-shell singlet. The initial scans from the MeO_4_Me minimum up to 3 Å O–O distance were done with tight resolution (40 steps, 0.04 Å interval) to capture any possible formation transition states or prereactive complexes. This scan range is shown as gray-shaded enlargements in Figure 1. The remaining range, from 3 to 15 Å, was scanned in 20 steps, with 0.63 Å intervals.

CASSCF(6e,6o) results show a smooth PES all the way to 3 Å, without indications of saddle points or prereactive complexes. CASSCF(10e,8o) and CASSCF(22e,14o) results both suggest a saddle point for tetroxide formation. The CASSCF(10e,8o) surface (up to 3 Å) appears different from the other CASSCF surfaces. Additionally, it is not as smooth as the other surfaces, which suggests instabilities in the (10e,8o) active space. We inspected the evolution of active space orbitals in the scans with the (10e,8o) active space and verified that some CAS orbitals had rotated with inactive orbitals. Specifically, the lonepair/π orbitals localized in the MeOO···OOMe bond had rotated with π orbitals localized in MeO···OO···OMe bonds. These rotations resulted in the artificial relaxation of the CASSCF energy. The rotations explain why the CASSCF(10e,8o) surface up to 3 Å separation appears different from the two other CASSCF surfaces. Due to the rotations, the relative energies of MeO_4_Me and isolated MeO_2_ radicals are not meaningful because the (10e,8o) active spaces are effectively different in these stationary points.

The largest active space, (22e,14o), was constructed to circumvent the issues with the (10e,8o) active space. Initially, we tried to enlarge the (10e,8o) active space by adding only the problematic inactive orbitals that caused the aforementioned rotations but were unable to obtain a stable active space. We thus decided to add all oxygen valence orbitals, except the two C–O σ-bonding orbitals, to the active space, yielding the (22e,14o) active space.

The tail of the potential, from 3 to 15 Å, appears very similar in all three CASSCF surfaces, both in terms of shape and relative energies (2–2.5 kcal mol^–1^ increase). The increase in energy after 6 Å coincides with the two peroxyl groups starting to rotate away from each other. The difference between the active spaces is most apparent in the dispersion interaction region, up to around 3 Å separation. The relative energy difference between isolated peroxyl radicals and tetroxide minimum varies between the used active spaces. The CASSCF(6e,6o) results suggest a 20 kcal mol^–1^ energy difference (Figure 1a), while the CASSCF(10e,8o) and CASSCF(22e,14o) results show smaller energy differences, 3 and 6.5 kcal mol^–1^, respectively (Figure 1b,c). All of these CASSCF relative energies significantly deviate from the CC relative energy.

The interaction between peroxyl radicals is affected by dynamical correlation. Therefore, we calculated CASPT2 single-point energy corrections (dotted lines in Figure 1) to each PES optimized at the CASSCF level. The CASPT2 corrections increase the relative total energy of CASSCF(10e,8o) and CASSCF(22e,14o), while lowering it in CASSCF(6e,6o). The increase in relative energy is expected because the CASSCF method is effectively devoid of any treatment of weak correlation, apart from the full configuration interaction within the CAS. The decrease in relative energy when adding perturbational correction might be due to the (6e,6o) active space not being large enough to capture all relevant static correlations and, thus, the effect of the CASPT2 correction is erratic.

With (10e,8o) and (22e,14o) active spaces, the PT2 corrections appear to stabilize the prereactive complex. The CASPT2 corrections on the CASSCF(10e,8o) surface did not yield a continuous potential. This is likely due to the aforementioned orbital rotations. The CASPT2 energy is not invariant with respect to rotations between inactive and active orbital space.^72^ Because the CASPT2 corrections to CASSCF(6e,6o) surface are erratic and the (10e,8o) active space is unstable due to rotations, further benchmarking was done mainly with the (22e,14o) active space.

**Figure 2:**
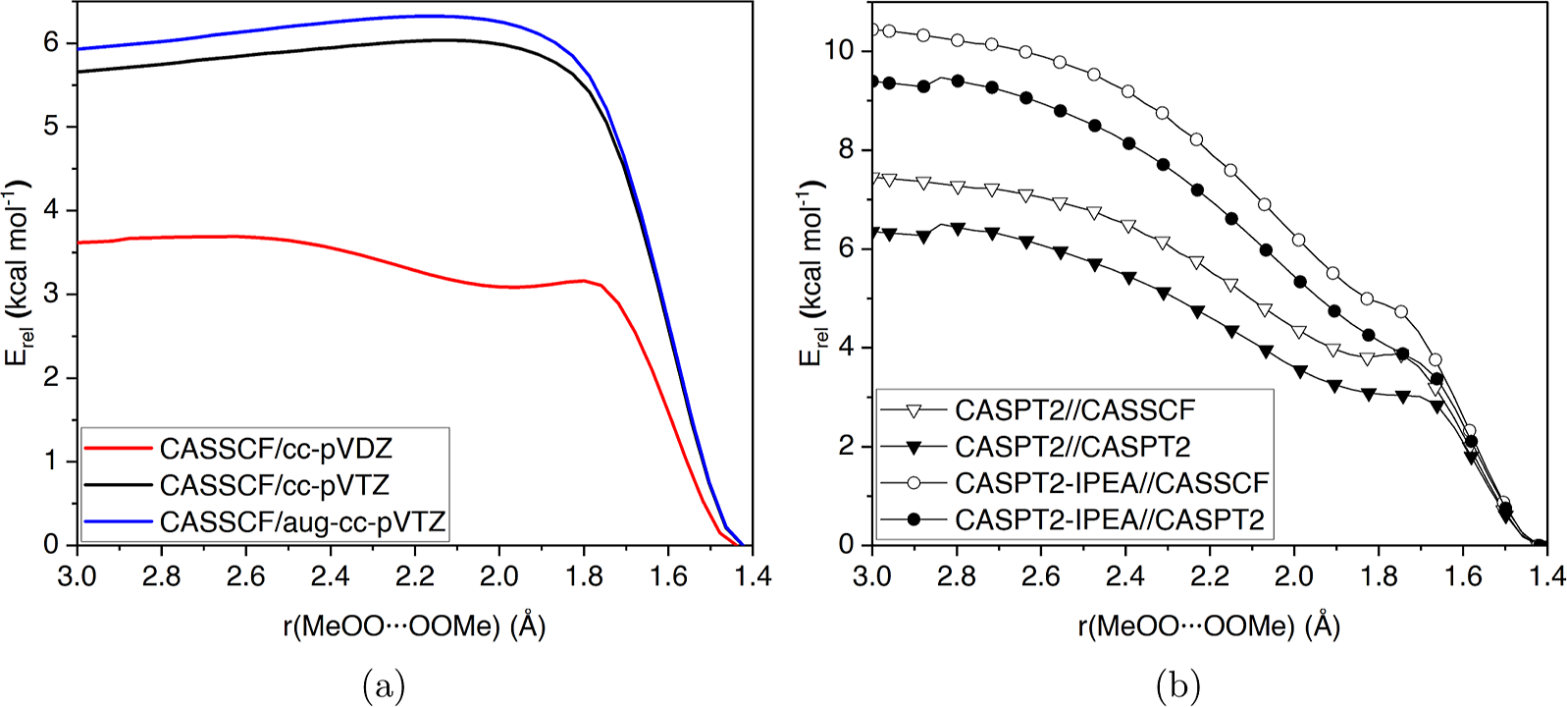
Relaxed scan of MeOO···OOMe bond length: (a) CASSCF(22e,14o) with various basis sets, and (b) CASPT2 (▽) and CASPT2-IPEA (○) corrections on geometries optimized with either CASSCF (hollow) or CASPT2 (solid), with cc-pVTZ basis set.

Next, we checked the effect of increasing the basis set size from cc-pVDZ to cc-pVTZ or aug-cc-pVTZ, and using an IPEA shift in the CASPT2 correction (Figure 2). We limited these scans to the dispersion region (from the MeO_4_Me minimum up to 3 Å O–O distance, 40 steps, 0.04 Å intervals), where we noticed the largest deviations in the preceding PES calculations. The IPEA shift value of 0.20 au was decided based on an internal benchmark, as discussed in the computational methods section and in Supporting Information (Section S4).

The CASSCF surfaces calculated with cc-pVTZ and aug-cc-pVTZ appear very similar, meaning that the diffuse functions in the basis set have very little effect on the results. On the other hand, results with the cc-pVDZ basis set deviate substantially from the two larger basis sets, suggesting that at least a triple-ζ quality basis set is needed for studying the formation reaction.

At the CASSCF level, the prereactive complex at around 2.0 Å reaction disappeared when the basis set was changed from cc-pVDZ to cc-pVTZ or aug-cc-pVTZ (Figure 2a). This suggests that the prereactive complex might be an artifact due to basis set superposition error (BSSE). Therefore, we calculated a counterpoise correction (CP) to the interaction energy of the prereactive complex at CASSCF(22e,14o)/cc-pVDZ level using the Boys–Bernardi scheme.^73^ The CP-correction decreased the interaction energy (MeO_2_ + MeO_2_ → MeO_2_···MeO_2_) from −3.27 to −2.03 kcal mol^–1^, which shifts the prereactive complex to a higher total energy than the formation transition state predicted at this level of theory. This result is in line with the hypothesis of BSSE. We were not able to do CP correction for the CASPT2 results since CASPT2 is not a size-consistent method.

The CASPT2-corrected CASSCF surface shows a shallow prereactive complex and a saddle point (Figure 2b, hollow triangles). Adding IPEA correction destabilizes the prereactive complex, and the resulting surface does not have a saddle point but instead a turning point at around 1.8 Å the O–O distance (Figure 2b, hollow circles). This turning point likely corresponds to bond formation.

The CASSCF surfaces (Figure 2a) look very different from the CASPT2- or CASPT2-IPEA-corrected CASSCF surfaces (Figure 2b, hollow symbols). To see how much dynamical correlation affects the optimized geometries and energies in the formation reaction pathway, we calculated surface scans also with CASPT2 geometry optimizations (Figure 2b, solid triangles), using the cc-pVTZ basis set. The CASPT2-optimized surfaces were also corrected with CASPT2-IPEA (Figure 2b, solid circles). The CASPT2-optimized surfaces look generally similar to the CASPT2-corrected CASSCF surfaces, suggesting that either methodology can be used for geometry optimizations when studying the formation reaction pathway.

Finally, we elongated and froze the distance between the peroxyl radicals to 30 Å and carried out constrained geometry optimizations at the CASSCF/cc-pVTZ and CASPT2/cc-pVTZ levels of theory, using all studied active spaces. The 30 Å separation was used to remove all attractive interactions between the radicals and better simulate the energy difference between infinitely separated MeO_2_ versus MeO_4_Me, to get descriptions comparable to the CC benchmark. Results from these calculations are shown in Table 2, in terms of the energy difference relative to MeO_4_Me. These results show that with the (22e,14o) active space, the relative energy difference approaches systematically the CC-benchmark value (14–15 kcal mol^–1^), when higher-level corrections are applied. Additionally, with the (22e,14o) active space, it does not matter whether the geometries are optimized at CASSCF or CASPT2 level because the CASPT2 (and CASPT2-IPEA) relative energies are almost identical in both approaches. Therefore, the CASPT2(22e,14o)-IPEA(0.20) method appears to be a good choice for investigating the formation reaction.

**Table 2:** Relative Energy Difference of Two 30 Å Separated MeO_2_ Radicals and MeO_4_Me Minimum, in kcal mol^–1^

method^a^	(6e,6o)	(10e,8o)^b^	(22e,14o)
CASSCF/cc-pVTZ	21.08	2.99	7.29
CASPT2//CASSCF	18.10	8.39	11.61
CASPT2-IPEA//CASSCF	21.75	12.12	14.97
CASPT2/cc-pVTZ	13.83	9.36	11.63
CASPT2-IPEA//CASPT2	17.77	12.98	14.99

a Reference values for relative energy differences: W3X-L//ωB97X-D3/aug-cc-pVTZ: 14.42 kcal mol^–1^, CCSD(T)/CBS//ωB97X-D3/aug-cc-pVTZ: 15.05 kcal mol^–1^.

b These values have a substantial uncertainty due to orbital rotations.

Overall, it is evident that the benchmarked variables, including the active space composition, dynamical correlation method, the basis set, and to a lesser extent the geometry optimization method, all have an appreciable effect on the formation reaction pathway, in terms of both the shape of the PES and the relative energies.

### 3.3 Decomposition Reaction, MeO_4_Me → MeO + MeO + O_2_

For the decomposition reaction of MeO_4_Me, we first calculated the relative energy difference of the tetroxide and the decomposition products, MeO + MeO + O_2_. To do this, we prepared a supermolecular calculation, in which the MeO radicals were separated from the O_2_ molecule symmetrically by 30 Å, followed by geometry optimization with the distance constraints, similar to what was done in the formation pathway calculations. We did these calculations with both CASSCF and CASPT2 levels of theory, using all three active spaces, with the cc-pVTZ basis set (Table 3).

**Table 3:** Energy Difference of Two 30 Å Separated MeO Radicals and O_2_ Relative to MeO_4_Me Minimum, in kcal mol^–1^

method^a^	(6e,6o)	(10e,8o)	(22e,14o)
CASSCF/cc-pVTZ	26.98	–12.21	–5.73
CASPT2//CASSCF	–5.46	12.00	16.73
CASPT2-IPEA//CASSCF	3.18	17.80	22.56
CASPT2/cc-pVTZ	–11.06	13.25	16.93
CASPT2-IPEA//CASPT2	–0.24	19.13	22.88

a Reference values for relative energy differences: W3X-L//ωB97X-D3/aug-cc-pVTZ: 22.87 kcal mol^–1^, CCSD(T)/CBS//ωB97X-D3/aug-cc-pVTZ: 23.01 kcal mol^–1^.

Results from these calculations show that only the CASPT2-IPEA corrected single-point energies with the (22e,14o) active space are able to accurately reproduce the relative energies calculated in the CC//DFT-benchmark. As for the formation reaction, it appears that geometries have only a minor effect on relative energies when the (22e,14o) active space is used. Thus, further calculations were done only with the (22e,14o) active space. Additionally, we calculated single-point energy corrections on the CASPT2(22e,14o)/cc-pVTZ optimized stationary structures to investigate the effect of various basis sets and dynamic correlation methods on the relative energies (Supporting Information Section S5). These results show that increasing the basis set size beyond cc-pVTZ or adding diffuse functions to the basis set has only a minor effect on the relative energies. In addition, the tested dynamic correlation methods (CASPT2, NEVPT2, and MRCISD + Q) other than CASPT2-IPEA are not able to reproduce the CC//DFT-benchmark results. The CASPT2, MRCISD + Q, and NEVPT2 results deviated from the CC//DFT benchmark by 3, 6, and 11 kcal mol^–1^, respectively.

**Figure 3:**
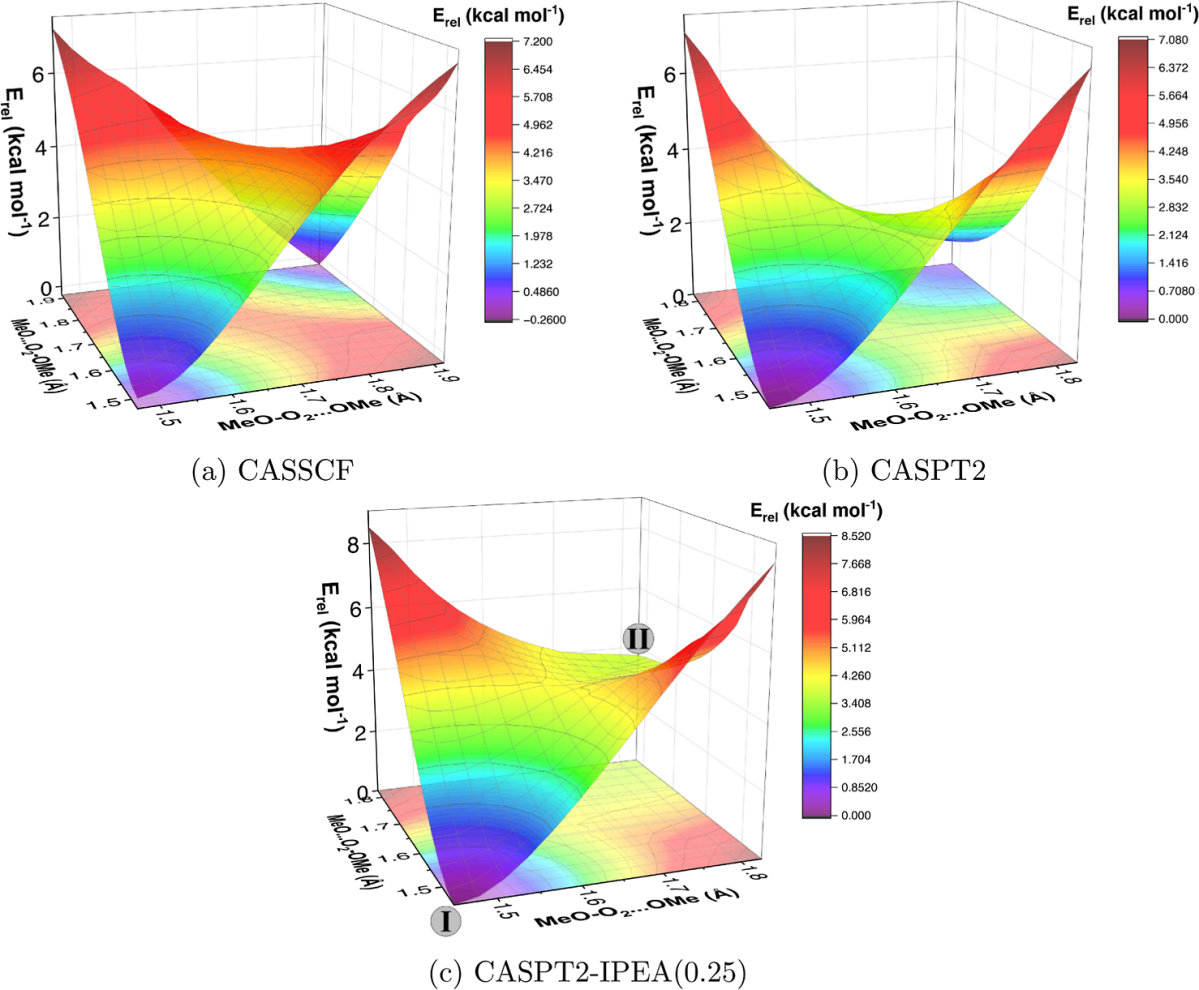
Relaxed 2-D scans along the two MeO···O bonds in MeO_4_Me calculated at (a) CASSCF, (b) CASPT2, and (c) CASPT2-IPEA(0.25) levels using (22e,14o) active space and cc-pVDZ basis set. The **I** corresponds to MeO_4_Me minimum in each graph, and **II** corresponds to the postreaction complex MeO···O_2_···MeO, as illustrated in (c).

Next, we studied the decomposition mechanism of the tetroxide. The decomposition may occur via two pathways. The first mechanism is that both MeO···O bonds in MeO_4_Me are broken simultaneously, producing two MeO and O_2_. Another possible decomposition mechanism occurs in two sequential reactions, where one O–O bond breaks first, yielding MeO and a methyltrioxyl radical (MeO_3_), which can subsequently dissociate into MeO and O_2_. To investigate which mechanism is preferred, we performed two-dimensional PES scans in which both MeO···O bonds in MeO_4_Me were elongated (Figure 3). The PES scans were carried out using geometries optimized at four levels of theory: CASSCF, CASPT2, CASPT2-IPEA, and NEVPT2 (see Supporting Information Section S5 for NEVPT2 results), using the cc-pVDZ basis set. The CASPT2-IPEA scans were carried out using a shift value of 0.25 au, which differs from other CASPT2-IPEA calculations in the present work (0.20 au). The difference of 0.05 au in the IPEA shift should not affect the results significantly. The scans were done using 20 × 20 steps, from the MeO_4_Me minimum up to 2.0 Å MeO···O bond lengths with approximately 0.03 Å intervals. We were not able to obtain a full set of converged geometries after around 1.8–1.9 Å MeO···O separations; thus, the surfaces only show the fully converged sections of the scans. According to the scans, the CASSCF, CASPT2, and CASPT2-IPEA results all show that the minimum energy path in the decomposition follows the symmetric decomposition mechanism where both the O–O bonds are cleaved simultaneously.

**Figure 4:**
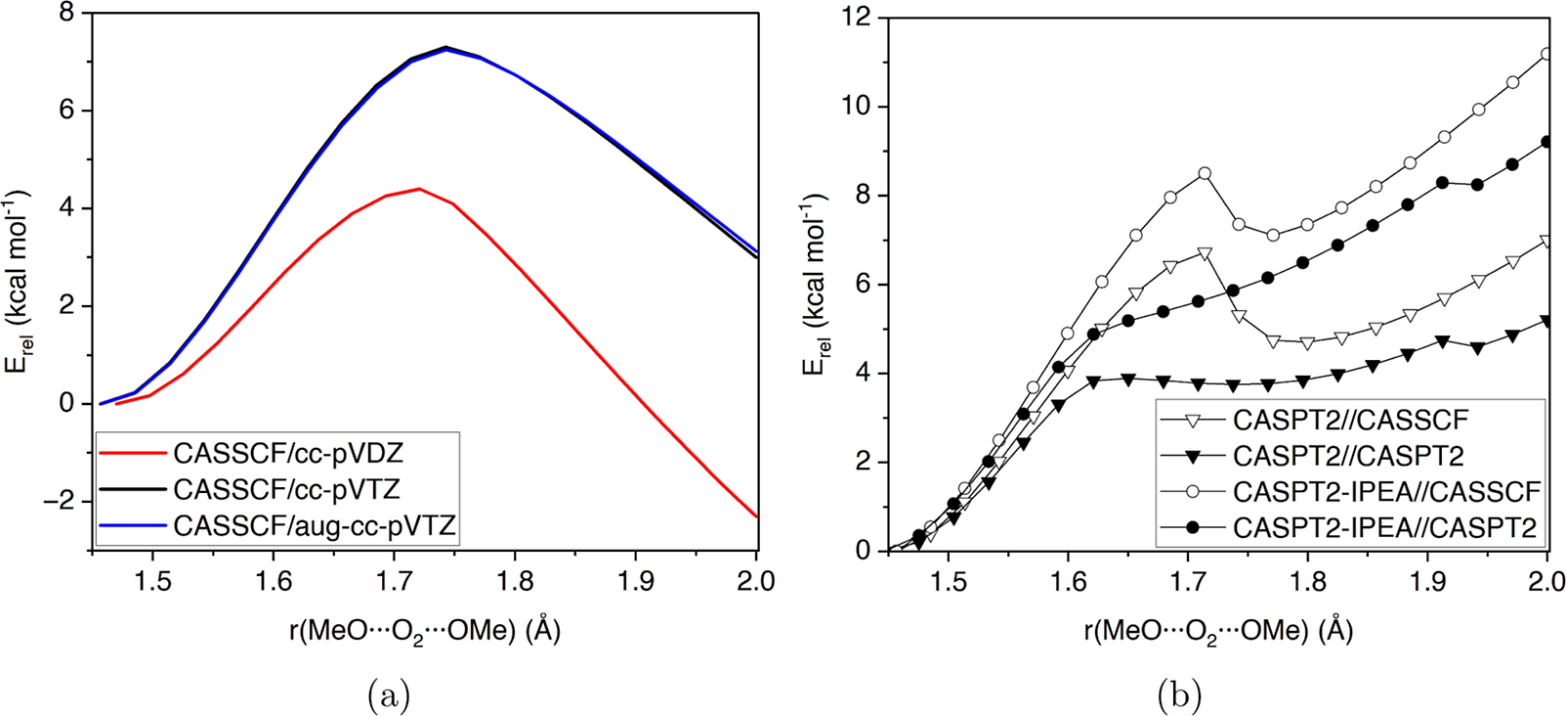
Relaxed scan of symmetric decomposition: (a) CASSCF(22e,14o) with various basis sets, and (b) CASPT2 (▽) and CASPT2-IPEA (○) corrections on geometries optimized with either CASSCF (hollow) or CASPT2 (solid), with cc-pVTZ basis set.

Then, we carried out relaxed surface scans in which we elongated both MeO···O bonds symmetrically up to 2 Å (starting from the MeO_4_Me minima, in 20 steps, with ca. 0.03 Å intervals) (Figure 4). We also tried to calculate the symmetric decomposition PES all the way to the isolated products, but after around 2.5 Å separation in the MeO···O_2_···OMe supermolecular system, we faced convergence issues, which are likely caused by the inclusion of too many weakly correlated orbitals in the active space. After the initial decomposition, when the three fragments are separated far enough from each other, the active space orbitals localized in the MeO radicals become very weakly correlated and show 2.0 natural orbital occupancies (1.0 for the radical). This leads to slow or oscillating CASSCF convergence (due to rotations within the active space). More stable scans would likely require the removal of weakly correlated orbitals from the active space, but this could simultaneously decrease the accuracy of the energies.

The symmetric scans were performed using geometries optimized with either the CASSCF or CASPT2 level. The CASSCF optimizations and subsequent calculations were carried out with the cc-pVDZ, cc-pVTZ, and aug-cc-pVTZ basis sets (Figure 4a), while CASPT2 optimizations were done with a cc-pVTZ basis set. We also tried to do geometry optimizations using the CASPT2-IPEA/cc-pVTZ level of theory. Likely, due to the aforementioned rotations within the active space, the CASPT2-IPEA gradient often started oscillating and did not yield stable solutions. The CASPT2-IPEA gradient is not invariant to orbital rotations within the active space.^74^

The CASSCF scans show a saddle point for decomposition at around 1.75 Å, followed by a continuous decrease in energy for the rest of the scanned distance (Figure 4a). When correcting the CASSCF curves with CASPT2 or CASPT2-IPEA single-point energy corrections (Figure 4b, hollow symbols), the saddle point is followed by shallow postreaction complex minimum and then an increase in energy. The CASPT2 optimized curve (Figure 4b, solid triangles) shows a very weakly bound postreaction complex, which is not present when IPEA-shift is employed (Figure 4b, solid circles). Although no saddle point is observed in the CASPT2-IPEA//CASPT2 potential energy surface, the turning point at around 1.7 Å may correspond to a free energy saddle point because there is a significant entropy benefit for breaking one molecule into three fragments.

Finally, we used the CASPT2(22e,14o)-IPEA/cc-pVTZ method for calculating total energies also for the bimolecular complexes of MeO···MeO and MeO···O_2_ that may form after the tetroxide decomposition, when either O_2_ or MeO is dissociated (Scheme 2). The geometries of these complexes were optimized using CASPT2(22e,14o)/cc-pVTZ. The geometry optimizations were done by constraining the distance of the dissociated fragment (O_2_ or MeO) from the remaining system by 30 Å, while other degrees of freedom were relaxed. Then, we calculated CASPT2(22e,14o)-IPEA/cc-pVTZ single-point energy corrections on the optimized geometries. These energies are shown relative to those of the tetroxide in Table 4.

**Table 4:** Relative Electronic Energies of MeO_2_ + MeO_2_, MeO_4_Me, MeO···MeO + O_2_, MeO···O_2_ + MeO, and MeO + MeO + O_2_, in kcal mol^–1^

method^a^	MeO_2_ + MeO_2_	MeO_4_Me	MeO···MeO^b^	MeO···O_2_^b^	MeO + MeO + O_2_
CC/CBS	15.05	0.00	19.63	22.20	23.01
W3X-L	14.42	0.00	19.30	18.80	22.87
CASPT2-IPEA	14.99	0.00	19.42	17.89	22.88

a CC/CBS and W3X-L single-point energy corrections calculated on ωB97X-D3/aug-cc-pVTZ optimized geometries. CASPT2-IPEA: CASPT2(22e,14o)-IPEA(0.20 au)/cc-pVTZ on CASPT2(22e,14o)/cc-pVTZ optimized geometries.

b MeO···MeO + ^3^O_2_ and MeO···O_2_ + ^2^MeO in CC/CBS and W3X-L. ^1^(MeO···MeO + O_2_) and ^1^(MeO···O_2_ + MeO) in CASPT2-IPEA.

The CASPT2 optimized structure of MeO···MeO is relatively similar to what is obtained with DFT methods, but the MeO···O_2_ geometry is notably different from the DFT structure (see Supporting Information Section S6). DFT methods suggest two minima for this system: covalently bound trioxyl radical (MeO_3_, MeO···OO bond length 1.50 Å) and the vdw-complex (MeO···O_2_, MeO···OO distance 3.18 Å), of which the latter is the global minimum structure. The CASPT2-optimized structure has a MeO–OO bond length of 1.87 Å, which is between the two DFT minima. The CASPT2 structure is likely more accurate than either of the DFT minima, because for the analogous hydrotrioxyl radical (HO_3_), CASPT2 method yields a structure more similar to the experimentally obtained structure than DFT or CCSD(T).^70,71^

In summary, we have shown that the PESs of both the formation and decomposition of MeO_4_Me largely depend on the level of theory. Of all tested multireference methods, the CASPT2(22e,14o)-IPEA(0.20)/cc-pVTZ is the only one able to reproduce the energetics found by the various coupled-cluster methods. We also found that the formation and decomposition transition states, and prereaction and postreaction complexes, are not present on the potential energy surface at the CASPT2(22e,14o)-IPEA(0.20)/cc-pVTZ level of theory. Further research is needed to see whether the observed features on the PES pertain to systems other than R = Me. Also, even though the MeO···O_2_···MeO complex does not appear to be a minimum on PES, it is still unclear whether the following postdecomposition reactions could occur directly from that state or if dissociation of one of three fragments is required for further reactions. Hopefully, further studies will elucidate these matters.

## 4 Conclusions

We benchmarked various coupled-cluster methods for calculating relative energies for the MeO_2_ + MeO_2_ → MeO_4_Me → MeO + MeO + O_2_ reactions. We used CCSD(T)/CBS and composite coupled-cluster methods W2X and W3X-L on ωB97X-D3/aug-cc-pVTZ optimized geometries. We found that the CCSD(T)/CBS and W2X and W3X-L composite methods yield similar relative energies, which confirms that coupled-cluster methods can be used for studying these stationary points. Furthermore, we used the same methods for calculating relative energies for the MeO···O_2_ and MeO···MeO complexes.

We evaluated multireference methods against the CC/CBS and W3X-L benchmarks by testing the effect of various active spaces, dynamical correlation methods, and basis sets. We found that the active space needs to be large, dynamical correlation corrections are important, and while at least a triple-ζ basis set is necessary, diffuse functions in basis sets have only a minor effect on relative energies. The optimized geometries of the MeO_2_ radical, the MeO radical, MeO_4_Me, and O_2_ are relatively insensitive toward the level of theory. Both CASSCF(22e,14o) and CASPT2(22e,14o) geometry optimizations yield similar structures. CASPT2-IPEA corrections are necessary for obtaining accurate relative energies, independent of the level of geometry optimization. We conclude that the combination of a (22e,14o) active space, CASPT2-IPEA(0.20 au) dynamical correlation correction on either CASSCF or CASPT2 optimized geometries, and the cc-pVTZ basis set is able to reproduce the relative energies of the stationary points in the CCSD(T)/CBS benchmark with excellent accuracy. We also want to emphasize that from all the tested multireference methods (CASPT2, NEVPT2, MRCISD + Q, CASPT2-IPEA), only the CASPT2-IPEA can reproduce the CC//DFT energetics, so this method is recommended for studying related reactions.

The CASPT2 optimized reaction surfaces suggest that the tetroxide formation is exothermic and its decomposition is endothermic. Possible saddle points, prereactive complexes, and postreaction complexes are sensitive to the level of theory. The CASPT2(22e,14o)-IPEA/cc-pVTZ method, which appears to be the most accurate of all tested multireference methods, suggests that the formation and decomposition reactions of MeO_4_Me do not have saddle points along the minimum energy path on the potential energy surface.

We also investigated the tetroxide decomposition mechanism. Results of two-dimensional relaxed surface scans, in which the two MeO···O bond lengths in MeO_4_Me were used as constrained reaction coordinates, suggest that the decomposition follows a pathway in which both of the MeO···O bonds are broken simultaneously. Results from scans, where both the MeO···O bonds are symmetrically elongated, show that after the decomposition CASSCF and CASPT2 surfaces appear qualitatively different. With CASSCF the decomposition is exothermic, while CASPT2 suggests endothermic decomposition.

We show with both the CASPT2(22e,14o)-IPEA and W3X-L methods that the MeO···O_2_ complex is more bound than the MeO···MeO complex, which is in contradiction to what has been believed so far. This result cannot be generalized to other systems, because larger alkoxyl radicals may have functional groups that increase intermolecular binding more in the RO···RO complexes, while binding with O_2_ likely is not affected as much.

Overall, we provide a detailed study of the formation and decomposition of MeO_4_Me. Further studies are needed for an accurate description of the various product formation channels after the initial decomposition of the tetroxide. It is likely that at least CASPT2 quality geometries are necessary for these reactions, due to the qualitative differences between CASSCF- and CASPT2-optimized surfaces discussed above. On the other hand, we found that CASSCF-optimized geometries can be used to calculate the relative energies of MeO_2_ + MeO_2_ → MeO_4_Me → MeO + MeO + O_2_, and to simulate the formation reaction pathway. CASSCF geometries with CASPT2 or CASPT2-IPEA energy corrections are much cheaper to calculate; therefore, such an approach can also be used to study larger RO_4_R (R larger than Me) systems.

## Data Availability

Quantum chemical calculation output files underlying the present study are available in “Supporting Information for Multireference and Coupled Cluster Study of Dimethyltetroxide (MeO_4_Me) Formation and Decomposition” at http://doi.org/10.5281/zenodo.10277012.

## Supporting Information Available

Contains visualizations of complete active spaces, additional 2D surface scans, PT2 correction diagnostics, IPEA-shift value benchmark, relative energies with various basis sets and methods, structure analysis of various studied stationary structures, and xyz-coordinates of relevant molecular structures (PDF)

## Footnotes

a Our use of the term unstable complex for the RO···O_2_···RO state implies that it may not exist as a stable intermediate complex on the potential energy surface. Our scans (see Figure 4b) indicate that MeO···O_2_···MeO complex is not a minimum on the potential energy surface.
