# Supplementary material for: Multireference and Coupled-Cluster Study of Dimethyltetroxide (MeO4Me) Formation and Decomposition
Source: J Phys Chem A. 2024 Feb 28;128(10):1825–36. doi: 10.1021/acs.jpca.3c08043 (PMC11465643; doi:10.1021/acs.jpca.3c08043)
Supplement: Supplementary file 1 — jp3c08043_si_001.pdf [file jp3c08043_si_001.pdf]

# Supporting Information for: Multireference and Coupled Cluster Study of Dimethyltetroxide ($MeO_4Me$) Formation and Decomposition

Vili-Taneli Salo,*,† Jing Chen,‡ Nino Runeberg,† Henrik G. Kjaergaard,‡ and Theo Kurtén*,†

†*Department of Chemistry, Faculty of Science, University of Helsinki, Helsinki FI-00014, Finland*

‡*Department of Chemistry, University of Copenhagen, 2100 Copenhagen, Denmark*

E-mail: vili-taneli.salo@helsinki.fi; theo.kurten@helsinki.fi

# Table of Contents


**S1 Complete active spaces visualized** **S6**

**S2 Two-dimensional relaxed surface scans** **S9**

**S3 PT2 diagnostics** **S10**

S3.1 $MeO_2 + MeO_2 \rightarrow MeO_4Me$ . . . . . . . . . . . . . . . . . . . . . . . . . . S12

S3.2 $MeO_4Me \rightarrow MeO + MeO + O_2$ . . . . . . . . . . . . . . . . . . . . . . . . S12

S3.3 CASPT2 energy denominator analysis . . . . . . . . . . . . . . . . . . . . . S13

**S4 Effect of IPEA shift to PT2 corrections and relative total energies** **S14**

**S5 Relative total energies with various basis sets and methods** **S17**

**S6 Structure analysis of various studied stationary points** **S19**

**S7 CC//DFT Benchmark** **S25**

**S8 DFT geometries** **S25**

S8.1 $\omega$B97X-D3/aug-cc-pVTZ . . . . . . . . . . . . . . . . . . . . . . . . . . . S25

S8.2 M06-2X/aug-cc-pVTZ . . . . . . . . . . . . . . . . . . . . . . . . . . . . . S28

**S9 CASSCF geometries** **S31**

S9.1 CAS(6e,6o)/cc-pVDZ . . . . . . . . . . . . . . . . . . . . . . . . . . . . . S31

S9.2 CAS(10e,8o)/cc-pVDZ . . . . . . . . . . . . . . . . . . . . . . . . . . . . S32

S9.3 CAS(22e,14o)/cc-pVDZ . . . . . . . . . . . . . . . . . . . . . . . . . . . . S34

S9.4 CAS(6e,6o)/cc-pVTZ . . . . . . . . . . . . . . . . . . . . . . . . . . . . . S35

S9.5 CAS(10e,8o)/cc-pVTZ . . . . . . . . . . . . . . . . . . . . . . . . . . . . S37

S9.6 CAS(22e,14o)/cc-pVTZ . . . . . . . . . . . . . . . . . . . . . . . . . . . . S38

S9.7 CAS(22e,14o)/aug-cc-pVTZ . . . . . . . . . . . . . . . . . . . . . . . . . . S40

**S10 CASPT2 geometries** **S41**

S10.1 CAS(6e,6o)/cc-pVDZ . . . . . . . . . . . . . . . . . . . . . . . . . . S41

S10.2 CAS(10e,8o)/cc-pVDZ . . . . . . . . . . . . . . . . . . . . . . . . . S43

S10.3 CAS(22e,14o)/cc-pVDZ . . . . . . . . . . . . . . . . . . . . . . . . S44

S10.4 CAS(6e,6o)/cc-pVTZ . . . . . . . . . . . . . . . . . . . . . . . . . . S46

S10.5 CAS(10e,8o)/cc-pVTZ . . . . . . . . . . . . . . . . . . . . . . . . . S47

S10.6 CAS(22e,14o)/cc-pVTZ . . . . . . . . . . . . . . . . . . . . . . . . S49

**References** **S51**

# List of Figures

S1 CAS(6e,6o) active orbitals. . . . . . . . . . . . . . . . . . . . . . . . . . . . S6

S2 CAS(10e,8o) active orbitals. . . . . . . . . . . . . . . . . . . . . . . . . . . S7

S3 CAS(22e,14o) active orbitals. . . . . . . . . . . . . . . . . . . . . . . . . . S8

S4 Relaxed 2-D scans along the two MeO···O bonds in $MeO_4Me$ calculated at a) CASSCF(6e,6o), b) CASSCF(10e,8o), c) CASSCF(22e,14o), d) CASPT2(6e,6o), e) CASPT2(10e,8o), and f) CASPT2(22e,14o), using the cc-pVDZ basis set. S9

S5 Relaxed 2-D scans along the two MeO···O bonds in $MeO_4Me$ calculated at a) NEVPT2(22e,14o), b) CASPT2(22e,14o)-IPEA(0.25), using the cc-pVDZ basis set. . . . . . . . . . . . . . . . . . . . . . . . . . . . . . . . . . . S10

S6 PT2 energy corrections and reference weights during symmetric $MeO_4Me$ decomposition. . . . . . . . . . . . . . . . . . . . . . . . . . . . . . . . . S13

S7 Effect of various IPEA shift values on relative total energies, calculated with CASPT2(22e,14o)/cc-pVTZ level of theory. Geometries for W3X-L energies optimized $\omega$B97X-D3/aug-cc-pVTZ. . . . . . . . . . . . . . . . . . . . . . S16

# List of Tables


S1 Second order perturbation energy correction diagnostics for $MeO_2$+$MeO_2$ $\longrightarrow$ $MeO_4Me$ $\longrightarrow$ MeO + MeO + $O_2$, with all used active spaces. . . . . . . . . . S11

S2 Denominator analysis of PT2 corrections of $MeO_2$ + $MeO_2$, $MeO_4Me$, and MeO + MeO + $O_2$ stationary points with all used active spaces. Obtained from CASPT2/cc-pVDZ calculations. . . . . . . . . . . . . . . . . . . . . . . . . S14

S3 Effect of IPEA shift to PT2 energy corrections and reference weights. Calculated at CASPT2(22e,14o)-IPEA/cc-pVTZ level of theory. . . . . . . . . . . S15

S4 Relative total energies (kcal mol$^{-1}$) with various basis sets and levels of theory.[a] Geometries optimized with CASPT2(22e,14o)/cc-pVTZ. . . . . . . . . . . . S17

S5 Effect of various active spaces and dynamical correlation methods on the calculated energies, relative to the $MeO_4Me$ in the total reaction $MeO_2$ + $MeO_2$ $\rightarrow$ $MeO_4Me$ $\rightarrow$ MeO + MeO + $O_2$. Geometries of the stationary points optimized with CASSCF using the corresponding active space and cc-pVTZ basis set, energies in kcal mol$^{-1}$. . . . . . . . . . . . . . . . . . . . . . . . . . . . S18

S6 Effect of various active spaces and dynamical correlation methods on the calculated energies, relative to the $MeO_4Me$ in the total reaction $MeO_2$ + $MeO_2$ $\rightarrow$ $MeO_4Me$ $\rightarrow$ MeO + MeO + $O_2$. Geometries of the stationary points optimized with CASPT2 and NEVPT2 using the corresponding active space and cc-pVTZ basis set, energies in kcal mol$^{-1}$. . . . . . . . . . . . . . . . . . . . . S19

S7 Structural parameters of $MeO_4Me$ with various levels of theory. . . . . . . . S21

S8 Structural parameters of $MeO_2$ with various levels of theory. . . . . . . . . . S22

S9 Structural parameters of MeO with various levels of theory. . . . . . . . . . . S23

S10 Structural parameters of $^3O_2$ with various levels of theory. . . . . . . . . . . S24

S11 Structural parameters of MeO...MeO with various levels of theory. . . . . . . S24

S12 Structural parameters of $MeO_3$ with various levels of theory. . . . . . . . . . S24

S13 Structural parameters of MeO...$O_2$ with various levels of theory. . . . . . . S24

S14 CC//DFT relative total energies of $MeO_2$+$MeO_2$, $MeO_4Me$, MeO...MeO+$O_2$, MeO...$O_2$+MeO, and MeO + MeO + $O_2$, in kcal mol$^{-1}$. . . . . . . . . . . . . S25

# S1 Complete active spaces visualized

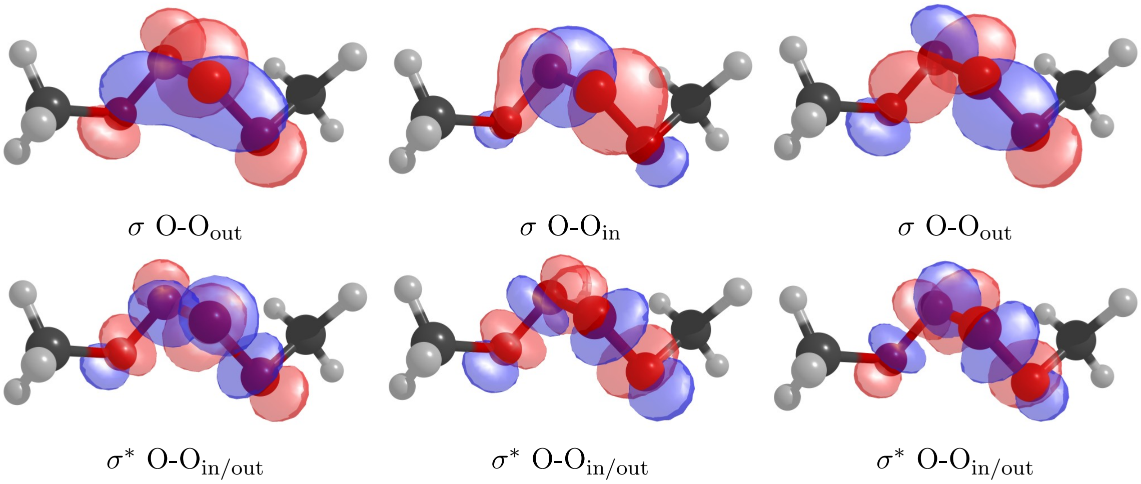


Figure S1: CAS(6e,6o) active orbitals.

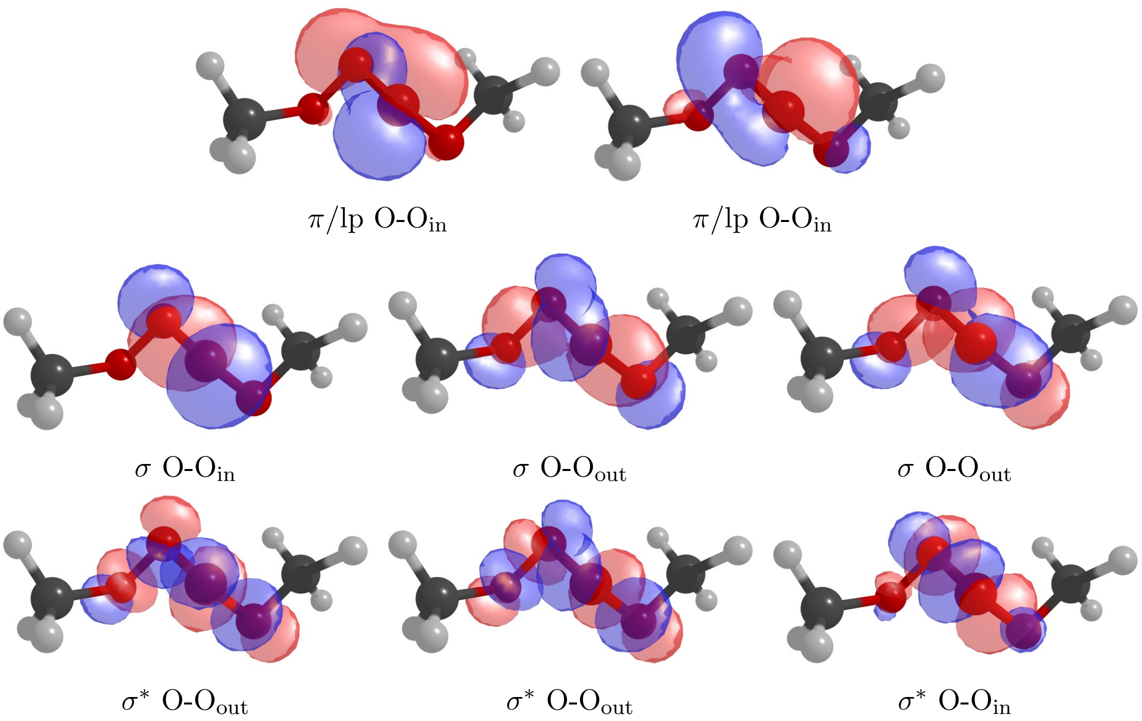


Figure S2: CAS(10e,8o) active orbitals.

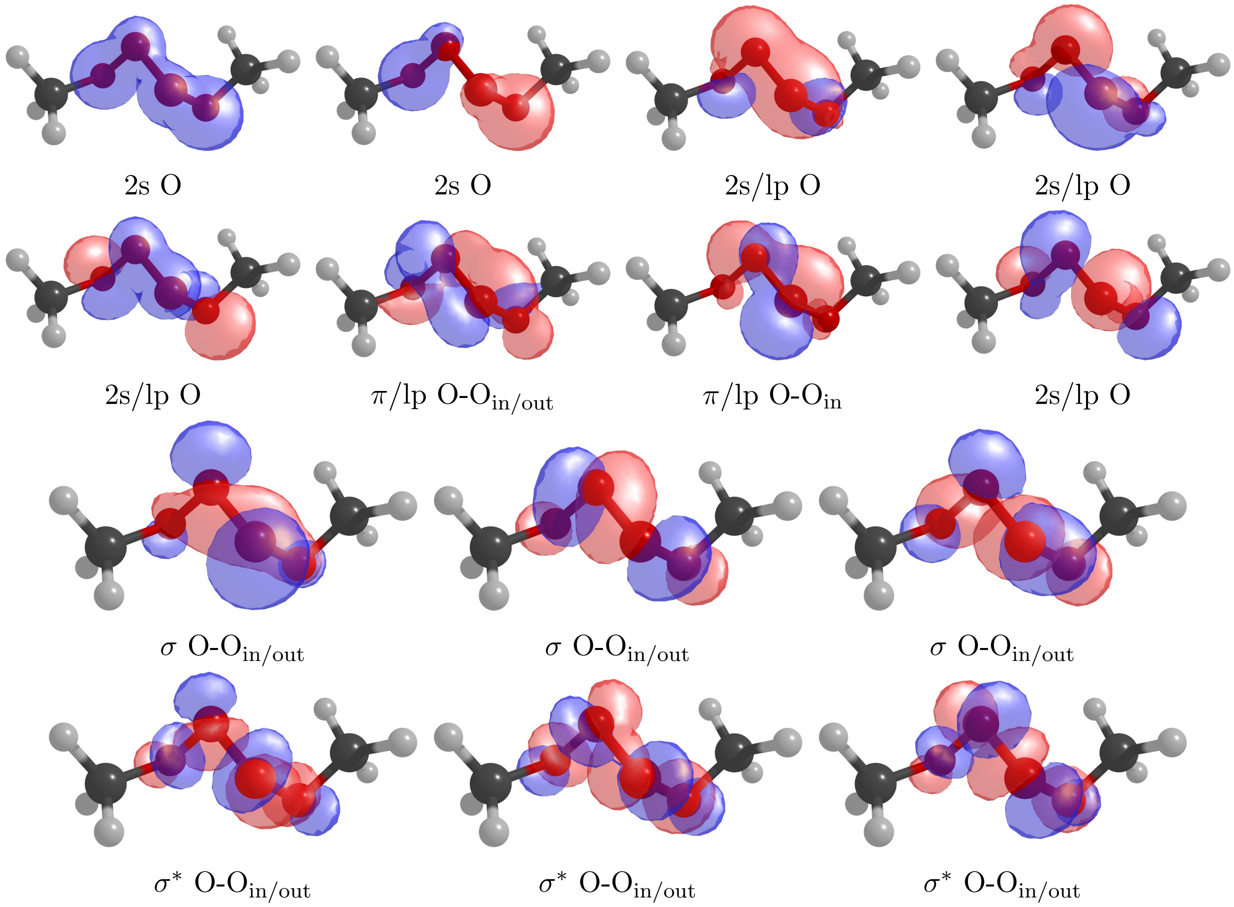


Figure S3: CAS(22e,14o) active orbitals.

# S2 Two-dimensional relaxed surface scans

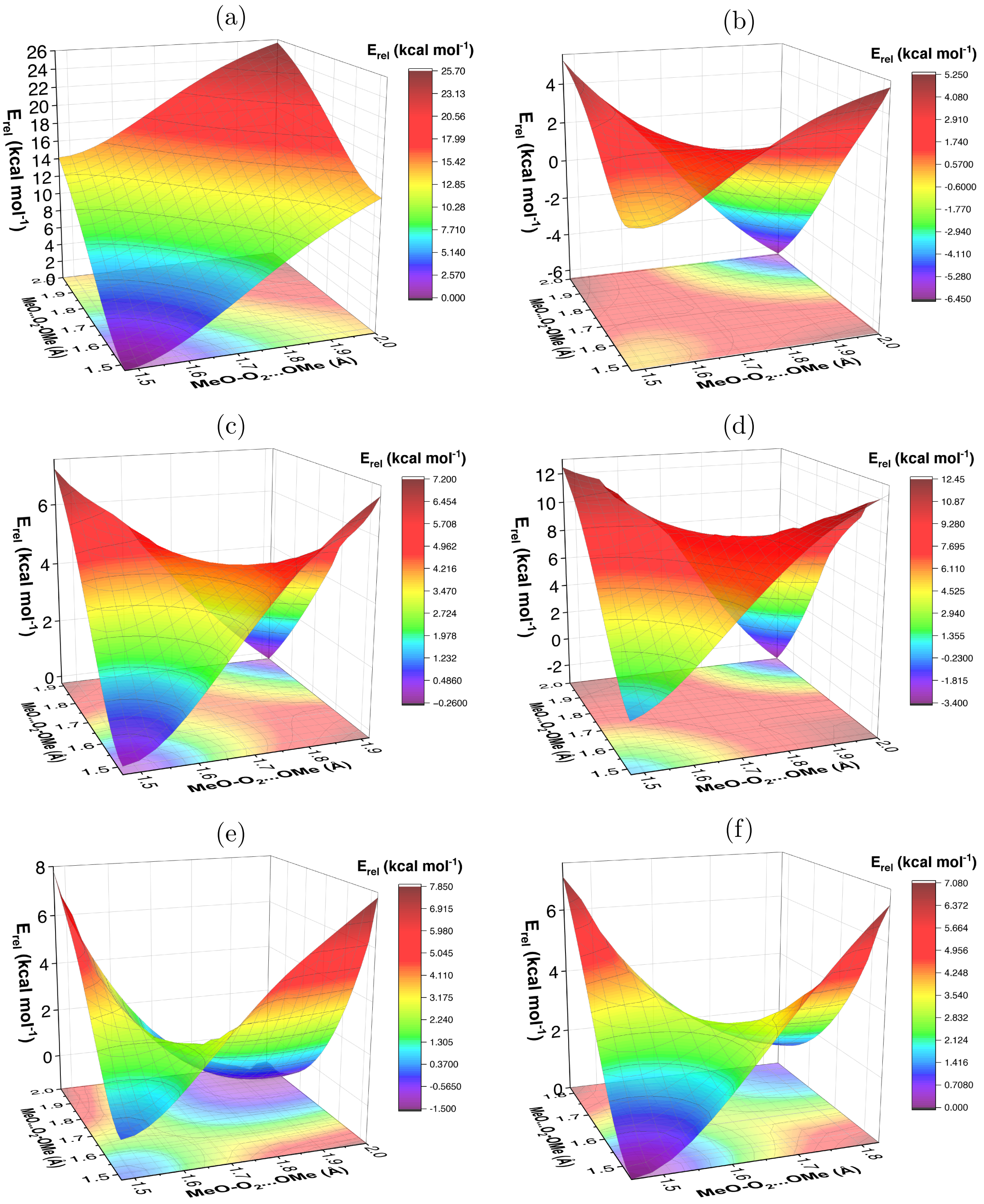


Figure S4: Relaxed 2-D scans along the two MeO···O bonds in $MeO_4Me$ calculated at a) CASSCF(6e,6o), b) CASSCF(10e,8o), c) CASSCF(22e,14o), d) CASPT2(6e,6o), e) CASPT2(10e,8o), and f) CASPT2(22e,14o), using the cc-pVDZ basis set.

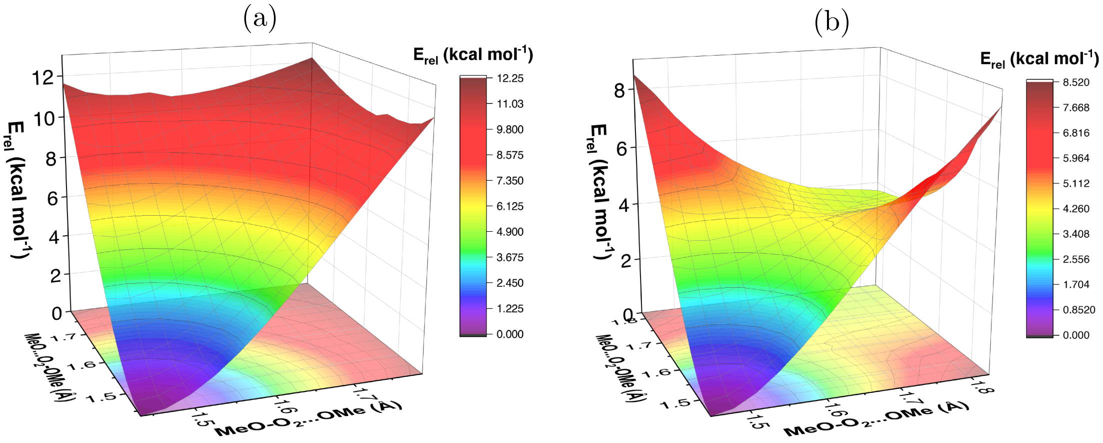


Figure S5: Relaxed 2-D scans along the two MeO···O bonds in $MeO_4Me$ calculated at a) NEVPT2(22e,14o), b) CASPT2(22e,14o)-IPEA(0.25), using the cc-pVDZ basis set.

# S3 PT2 diagnostics

To diagnose the perturbation corrections, we tabulated the reference weight (of the 0$^{th}$ order CAS wavefunction, RW), and the ratio of perturbation energy correction and corrected total energy (Table S1). The applicability of PT2 corrections can be approximated with the following formula,[1]

$$RW = (1 + \alpha)^{-\frac{N}{2}} \times 100\%$$

where $\alpha$ is a constant ranging from 0.01 to 0.02, and $N$ is the number of electrons excluding frozen core electrons. If the reference weight with a given active space is within this range (64.7-80.3% for the studied system), the PT2 correction is sufficiently small in comparison to 0$^{th}$ order CASSCF energy. Reference weights are relatively high, independent of the chosen active space. This means that PT2 correction may be applied to all of these CASSCF functions. Importantly, the ratio of perturbation correction and corrected total energy does not vary much between the extrema of PES. This shows that the PT2 corrections are of similar magnitude in all studied structures. There are some deviations from this trend, and those are discussed in detail below.

**Table S1: Second order perturbation energy correction diagnostics for $MeO_2 + MeO_2 \longrightarrow MeO_4Me \longrightarrow MeO + MeO + O_2$, with all used active spaces.**

| **CASPT2(6e,6o)/cc-pVDZ** | $MeO_2 + MeO_2$ | $MeO_4Me$ | $MeO + MeO + O_2$ |
|---|---|---|---|
| Reference Weight (%) | 78.82 | 79.68 | 75.66 |
| Energy Correction by PT2 (Eh) | -0.9233 | -0.9048 | -0.9820 |
| Ratio of energy correction and corrected total energy (%) | 0.2435 | 0.2385 | 0.2588 |
| Relative corrected total energies (kcal $mol^{-1}$) | 11.90 | 0.00 | -15.86 |
| with 0.25 IPEA shift | 15.90 | 0.00 | -4.20 |
| **CASPT2(10e,8o)/cc-pVDZ** | $MeO_2 + MeO_2$ | $MeO_4Me$ | $MeO + MeO + O_2$ |
| Reference Weight (%) | 79.94 | 79.87 | 80.33 |
| Energy Correction by PT2 (Eh) | -0.8911 | -0.8993 | -0.8648 |
| Ratio of energy correction and corrected total energy (%) | 0.2349 | 0.2370 | 0.2279 |
| Relative corrected total energies (kcal $mol^{-1}$) | 7.96 | 0.00 | 7.50 |
| with 0.25 IPEA shift | 11.43 | 0.00 | 13.18 |
| **CASPT2(22e,14o)/cc-pVDZ** | $MeO_2 + MeO_2$ | $MeO_4Me$ | $MeO + MeO + O_2$ |
| Reference Weight (%) | 80.17 | 80.18 | 80.55 |
| Energy Correction by PT2 (Eh) | -0.8841 | -0.8901 | -0.8588 |
| Ratio of energy correction and corrected total energy (%) | 0.2330 | 0.2346 | 0.2264 |
| Relative corrected total energies (kcal $mol^{-1}$) | 10.16 | 0.00 | 11.31 |
| with 0.25 IPEA shift | 13.43 | 0.00 | 17.12 |

## S3.1 $MeO_2 + MeO_2 \rightarrow MeO_4Me$

Reference weights for $MeO_2$ + $MeO_2$ and $MeO_4Me$ are similar for all used active spaces, which should also manifest as similar relative energies between said stationary points. This somewhat applies for CAS(6e,6o) and CAS(22e,14o), but not for CAS(10e,8o). Then again, relative energy difference in CAS(10e,8o) is not a meaningful result, as some active space orbitals have rotated with inactive orbitals when calculating the $MeO_2$ + $MeO_2$ structure. Because CASPT2 is not invariant with respect to rotations between inactive-active space, these CASPT2(10e,8o) energies are not comparable.

## S3.2 $MeO_4Me \rightarrow MeO + MeO + O_2$

A comparison of $MeO_4Me$ and MeO+MeO+$O_2$ reveals a sudden drop in reference weight for CAS(6e,6o), which suggests that such a CAS might not be good enough for describing the static electron correlation in the system. The failure of CAS(6e,6o) is further pronounced in the negative relative energy difference between MeO + MeO + $O_2$ and $MeO_4Me$. With CAS(10e,8o) and CAS(22e,14o) the reference weight increases. This makes sense as electron correlation in $O_2$ is largely described with (10e,8o) and (22e,14o) active spaces, and overall multireference character decreases as MeO fragments are being separated from $O_2$. Figure S6 shows the changes in reference weights and PT2 corrections during symmetric decomposition reaction of $MeO_4Me$. It appears that the perturbation correction is at its largest immediately after decomposition when the molecular complex system is stabilized by various weak interactions, such as intermolecular hydrogen bonds. None of the used active spaces include any C-H $\sigma$ or $\sigma$* orbitals, thus description of H-bonding are predominantly described *via* PT2 substitutions. Increasing the distance between MeO + MeO + $O_2$, and effectively cleaving the weak interactions, decreases the PT2 correction and increases the reference weights. In the Figure S6b, the discontinuation in the PT2 energies is due to conformational relaxation, which by visual inspection looks like a formation of hydrogen-bond between $O_2$ and MeO, further indicating that weak interactions contribute notably to PT2 correction en-

ergy. The difference in reference weights between the two used basis sets is logical, because the used active spaces are identical. The cc-pVTZ is larger basis set than cc-pVDZ, which means that CAS(22e,14o)/cc-pVDZ describes more electron correlation within that basis than CAS(22e,14o)/cc-pVTZ. Similarly, the PT2 corrections are larger for cc-pVTZ than for cc-pVDZ, because for larger basis, there are more individual contributions to perturbation correction.

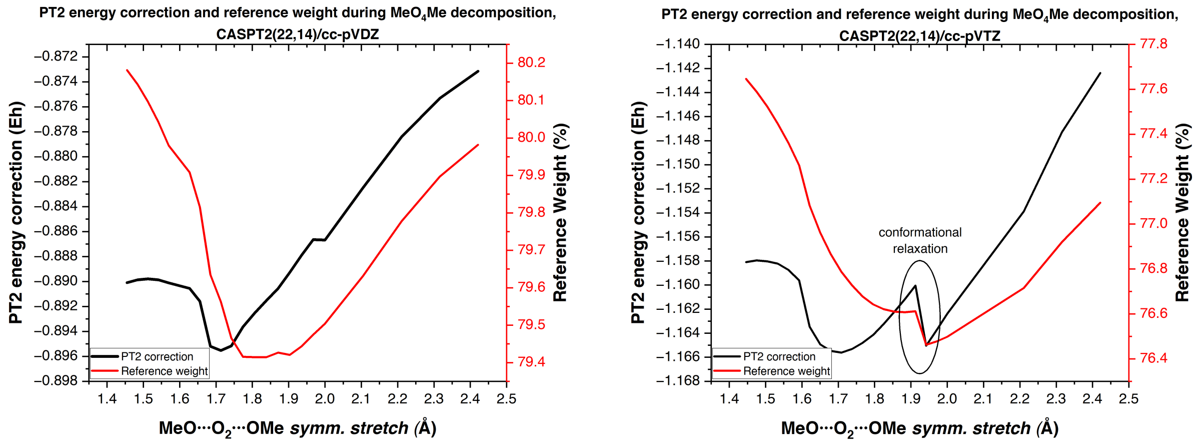


(a) CASPT2(22e,14o)/cc-pVDZ

(b) CASPT2(22e,14o)/cc-pVTZ

Figure S6: PT2 energy corrections and reference weights during symmetric $MeO_4Me$ decomposition.

## S3.3 CASPT2 energy denominator analysis

In Table S2, we have collected the smallest energy denominators in the PT2 energy correction terms in each of the eight different substitution classes for $MeO_2 + MeO_2$, $MeO_4Me$, and $MeO + MeO + O_2$ stationary points. No negative denominators were observed. Also, all denominators were relatively large, which indicates that intruder states are not an issue in the studied stationary points. Table 2 also shows the most contributing excitation class for each stationary point. With the smaller active spaces (6e,6o) and (10e,8o), the dominating PT2 contribution is from the internal→virtual excitations. With the (22e,14o) active space, most of the valence electrons are included in the active space and thus the active→virtual

excitations contribute the most (except for $MeO_4Me$ structure).

**Table S2: Denominator analysis of PT2 corrections of $\mathbf{MeO_2 + MeO_2}$, $\mathbf{MeO_4Me}$, and $\mathbf{MeO + MeO + O_2}$ stationary points with all used active spaces. Obtained from CASPT2/cc-pVDZ calculations.**[a]

| System | Substitution Class[b] | | | | | | | |
|---|---|---|---|---|---|---|---|---|
| | $\left\vert \begin{smallmatrix} A & B \\ I & J \end{smallmatrix} \right\rangle$ | $\left\vert \begin{smallmatrix} A & B \\ I & T \end{smallmatrix} \right\rangle$ | $\left\vert \begin{smallmatrix} T & A \\ I & J \end{smallmatrix} \right\rangle$ | $\left\vert \begin{smallmatrix} A & B \\ T & U \end{smallmatrix} \right\rangle$ | $\left\vert \begin{smallmatrix} T & U \\ I & J \end{smallmatrix} \right\rangle$ | $\left\vert \begin{smallmatrix} A & U \\ I & T \end{smallmatrix} \right\rangle$ | $\left\vert \begin{smallmatrix} V & A \\ T & U \end{smallmatrix} \right\rangle$ | $\left\vert \begin{smallmatrix} U & V \\ I & T \end{smallmatrix} \right\rangle$ |
| $MeO_2 + MeO_2$ | | | | | | | | |
| CAS(6e,6o) | **1.32** | 1.04 | 0.95 | 0.75 | 0.56 | 0.56 | 0.28 | 0.18 |
| CAS(10e,8o) | **1.31** | 1.06 | 0.90 | 0.71 | 0.49 | 0.51 | 0.23 | 0.14 |
| CAS(22e,14o) | 1.59 | 1.20 | 1.17 | **0.71** | 0.76 | 0.64 | 0.22 | 0.26 |
| $MeO_4Me$ | | | | | | | | |
| CAS(6e,6o) | **1.36** | 1.46 | 1.38 | 1.57 | 1.42 | 0.48 | 0.64 | 0.57 |
| CAS(10e,8o) | **1.36** | 1.34 | 1.35 | 1.32 | 1.32 | 0.47 | 0.49 | 0.53 |
| CAS(22e,14o) | **1.57** | 1.42 | 1.56 | 1.28 | 1.53 | 0.56 | 0.44 | 0.62 |
| $MeO + MeO + O_2$ | | | | | | | | |
| CAS(6e,6o) | **1.29** | 1.04 | 0.88 | 0.79 | 0.46 | 0.60 | 0.35 | 0.19 |
| CAS(10e,8o) | **1.29** | 1.04 | 0.84 | 0.77 | 0.40 | 0.55 | 0.30 | 0.15 |
| CAS(22e,14o) | 1.55 | 1.17 | 1.10 | **0.77** | 0.66 | 0.67 | 0.29 | 0.27 |

[a] Bold values correspond to the substitution class with highest contribution to PT2 energy correction.
[b] The indices IJ, TUV, and AB denote internal, active, and virtual orbitals, respectively.

# S4 Effect of IPEA shift to PT2 corrections and relative total energies

Using an IPEA shift increases the values of the denominator expressions in the $2^{nd}$ order perturbation energy corrections. Thus individual PT2 corrections evaluate to smaller values, ultimately decreasing the ratio of PT2 corrections to corrected total energies, compared to canonical CASPT2. Because the IPEA shift is an empirical parameter, we analyzed how sensitive the obtained results are with respect to the shift value. See Table S3 for the effect of various IPEA shifts on PT2 energies and reference weights. The values go from no IPEA correction (identical to canonical CASPT2) to shift value of 0.5 a.u., through the generally recommended value of 0.25 a.u. for molecules consisting of main group elements. The IPEA

shift unsurprisingly has most pronounced effect on $MeO_2 + MeO_2$ and $MeO + MeO + O_2$ PT2 corrections, and this is due to the former having two unpaired electrons and latter having four.

**Table S3: Effect of IPEA shift to PT2 energy corrections and reference weights. Calculated at CASPT2(22e,14o)-IPEA/cc-pVTZ level of theory.**

| $\mathbf{MeO_2 + MeO_2}$ | **PT2 (Eh)** | **Reference Weight (%)** |
|---|---|---|
| NOIPEA | -1.151 | 77.48 |
| 0.1 | -1.147 | 77.70 |
| 0.2 | -1.143 | 77.87 |
| 0.25 | -1.141 | 77.94 |
| 0.3 | -1.139 | 78.02 |
| 0.4 | -1.136 | 78.16 |
| 0.5 | -1.133 | 78.29 |
| $\mathbf{MeO_4Me}$ | **PT2 (Eh)** | **Reference Weight (%)** |
| NOIPEA | -1.158 | 77.65 |
| 0.1 | -1.156 | 77.73 |
| 0.2 | -1.155 | 77.78 |
| 0.25 | -1.155 | 77.80 |
| 0.3 | -1.154 | 77.82 |
| 0.4 | -1.153 | 77.86 |
| 0.5 | -1.152 | 77.89 |
| $\mathbf{MeO + MeO + O_2}$ | **PT2 (Eh)** | **Reference Weight (%)** |
| NOIPEA | -1.122 | 77.79 |
| 0.1 | -1.115 | 78.10 |
| 0.2 | -1.109 | 78.36 |
| 0.25 | -1.107 | 78.48 |
| 0.3 | -1.104 | 78.59 |
| 0.4 | -1.099 | 78.80 |
| 0.5 | -1.094 | 79.00 |

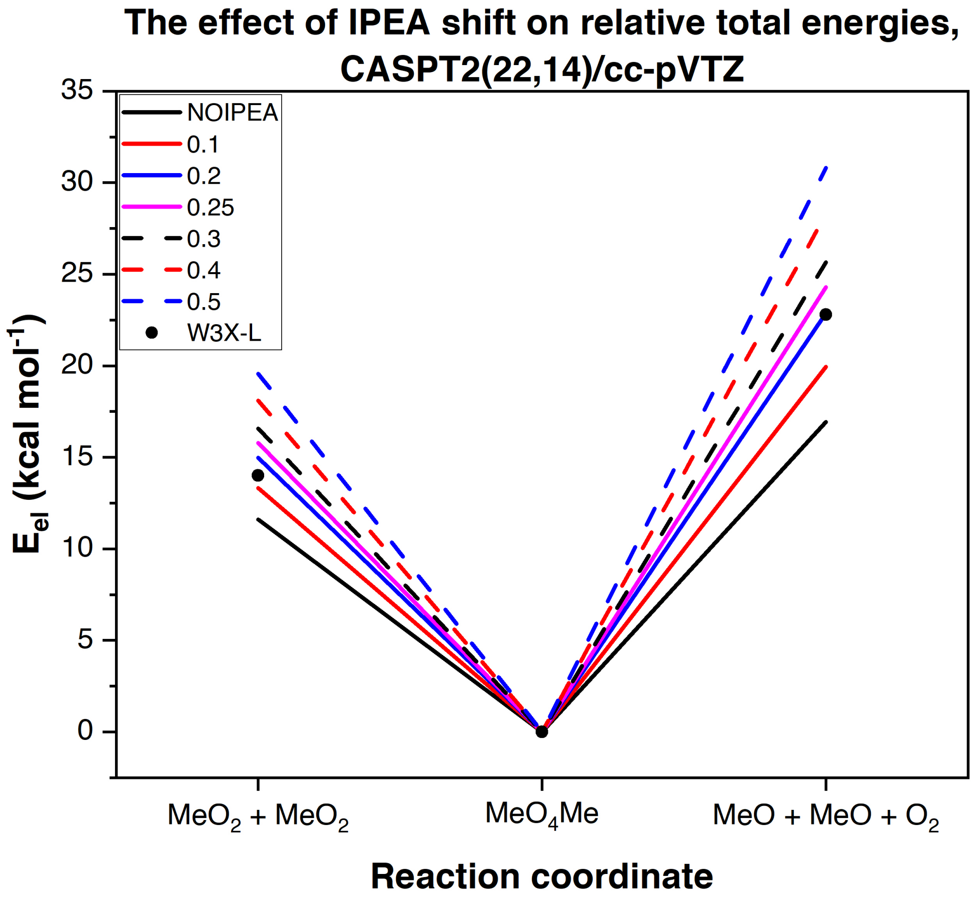


Figure S7: Effect of various IPEA shift values on relative total energies, calculated with CASPT2(22e,14o)/cc-pVTZ level of theory. Geometries for W3X-L energies optimized $\omega$B97X-D3/aug-cc-pVTZ.

# S5 Relative total energies with various basis sets and methods

**Table S4: Relative total energies (kcal mol$^{-1}$) with various basis sets and levels of theory.[a] Geometries optimized with CASPT2(22e,14o)/cc-pVTZ.**

| cc-pVDZ | $MeO_2 + MeO_2$ | $MeO_4Me$ | $MeO + MeO + O_2$ | aug-cc-pVDZ | $MeO_2 + MeO_2$ | $MeO_4Me$ | $MeO + MeO + O_2$ |
|---|---|---|---|---|---|---|---|
| CASSCF | 6.35 | 0.00 | -8.55 | CASSCF | 5.95 | 0.00 | -8.47 |
| CASPT2 | 10.11 | 0.00 | 11.24 | CASPT2 | 10.70 | 0.00 | 13.56 |
| NEVPT2 | 16.84 | 0.00 | 27.91 | NEVPT2 | 18.97 | 0.00 | 32.23 |
| MRCI+Q | 7.45 | 0.00 | 7.19 | MRCI+Q | 15.84 | 0.00 | 14.26 |
| CASPT2-IPEA | 12.77 | 0.00 | 15.96 | CASPT2-IPEA | 14.13 | 0.00 | 19.46 |
| cc-pVTZ | $MeO_2 + MeO_2$ | $MeO_4Me$ | $MeO + MeO + O_2$ | aug-cc-pVTZ | $MeO_2 + MeO_2$ | $MeO_4Me$ | $MeO + MeO + O_2$ |
| CASSCF | 7.31 | 0.00 | -5.85 | CASSCF | 7.09 | 0.00 | -6.27 |
| CASPT2 | 11.61 | 0.00 | 16.93 | CASPT2 | 11.93 | 0.00 | 18.03 |
| NEVPT2 | 19.50 | 0.00 | 34.75 | NEVPT2 | 20.39 | 0.00 | 36.63 |
| MRCI+Q | 17.63 | 0.00 | 19.86 | MRCI+Q | -[b] | -[b] | -[b] |
| CASPT2-IPEA | 14.99 | 0.00 | 22.89 | CASPT2-IPEA | 15.58 | 0.00 | 24.39 |
| cc-pVQZ | $MeO_2 + MeO_2$ | $MeO_4Me$ | $MeO + MeO + O_2$ | aug-cc-pVQZ | $MeO_2 + MeO_2$ | $MeO_4Me$ | $MeO + MeO + O_2$ |
| CASSCF | 6.76 | 0.00 | -6.79 | CASSCF | 6.66 | 0.00 | -7.03 |
| CASPT2 | 11.17 | 0.00 | 17.11 | CASPT2 | 11.43 | 0.00 | 17.84 |
| NEVPT2 | 19.50 | 0.00 | 35.50 | NEVPT2 | 19.96 | 0.00 | 36.50 |
| MRCI+Q | -[b] | -[b] | -[b] | MRCI+Q | -[b] | -[b] | -[b] |
| CASPT2-IPEA | 14.77 | 0.00 | 23.42 | CASPT2-IPEA | 15.13 | 0.00 | 24.31 |

[a] The (22e,14o) active space was used in all calculations.
[b] MRCI+Q too expensive with (22e,14o) active space and this basis set.

**Table S5: Effect of various active spaces and dynamical correlation methods on the calculated energies, relative to the $MeO_4Me$ in the total reaction $MeO_2$ + $MeO_2$ → $MeO_4Me$ → MeO + MeO + $O_2$. Geometries of the stationary points optimized with CASSCF using the corresponding active space and cc-pVTZ basis set, energies in kcal mol$^{-1}$.**

| CASSCF(6e,6o)/cc-pVTZ opt. geom. | $MeO_2 + MeO_2$ | $MeO_4Me$ | MeO + MeO + $O_2$ |
|---|---|---|---|
| CASSCF(6e,6o)/cc-pVTZ | 21.08 | 0.00 | 26.98 |
| CASPT2(6e,6o)/cc-pVTZ | 18.10 | 0.00 | -5.46 |
| CASPT2(6e,6o)-IPEA(0.20)/cc-pVTZ | 21.75 | 0.00 | 3.25 |
| NEVPT2(6e,6o)/cc-pVTZ | 23.04 | 0.00 | 12.49 |
| MRCISD(6e,6o)+Q/cc-pVTZ | 31.49 | 0.00 | 23.26 |
| CASSCF(10e,8o)/cc-pVTZ opt. geom. | $MeO_2 + MeO_2$ | $MeO_4Me$ | MeO + MeO + $O_2$ |
| CASSCF(10e,8o)/cc-pVTZ | 2.99 | 0.00 | -12.21 |
| CASPT2(10e,8o)/cc-pVTZ | 8.39 | 0.00 | 12.00 |
| CASPT2(10e,8o)-IPEA(0.20)/cc-pVTZ | 12.12 | 0.00 | 17.75 |
| NEVPT2(10e,8o)/cc-pVTZ | 11.43 | 0.00 | 24.58 |
| MRCISD(10e,8o)+Q/cc-pVTZ | 17.55 | 0.00 | 24.13 |
| CASSCF(22e,14o)/cc-pVTZ opt. geom. | $MeO_2 + MeO_2$ | $MeO_4Me$ | MeO + MeO + $O_2$ |
| CASSCF(22e,14o)/cc-pVTZ | 7.29 | 0.00 | -5.73 |
| CASPT2(22e,14o)/cc-pVTZ | 11.61 | 0.00 | 16.73 |
| CASPT2(22e,14o)-IPEA(0.20)/cc-pVTZ | 14.97 | 0.00 | 22.56 |
| NEVPT2(22e,14o)/cc-pVTZ | 19.27 | 0.00 | 34.32 |
| MRCISD(22e,14o)+Q/cc-pVTZ | 17.24 | 0.00 | 20.25 |

**Table S6: Effect of various active spaces and dynamical correlation methods on the calculated energies, relative to the $MeO_4Me$ in the total reaction $MeO_2$+$MeO_2$ → $MeO_4Me$ → MeO + MeO + $O_2$. Geometries of the stationary points optimized with CASPT2 and NEVPT2 using the corresponding active space and cc-pVTZ basis set, energies in kcal mol$^{-1}$.**

| CASPT2(6e,6o)/cc-pVTZ opt. geom. | $MeO_2$ + $MeO_2$ | $MeO_4Me$ | MeO + MeO + $O_2$ |
|---|---|---|---|
| CASPT2(6e,6o)/cc-pVTZ | 13.83 | 0.00 | -11.06 |
| CASPT2(6e,6o)-IPEA(0.20)/cc-pVTZ | 17.77 | 0.00 | -0.24 |
| CASPT2(10e,8o)/cc-pVTZ opt. geom. | $MeO_2$ + $MeO_2$ | $MeO_4Me$ | MeO + MeO + $O_2$ |
| CASPT2(6e,6o)/cc-pVTZ | 9.36 | 0.00 | 13.25 |
| CASPT2(6e,6o)-IPEA(0.20)/cc-pVTZ | 12.98 | 0.00 | 19.13 |
| CASPT2(22e,14o)/cc-pVTZ opt. geom. | $MeO_2$ + $MeO_2$ | $MeO_4Me$ | MeO + MeO + $O_2$ |
| CASPT2(22e,14o)/cc-pVTZ | 11.63 | 0.00 | 16.93 |
| CASPT2(22e,14o)-IPEA(0.20)/cc-pVTZ | 14.99 | 0.00 | 22.88 |
| NEVPT2(6e,6o)/cc-pVTZ opt. geom. | $MeO_2$ + $MeO_2$ | $MeO_4Me$ | MeO + MeO + $O_2$ |
| NEVPT2(6e,6o)/cc-pVTZ | 19.59 | 0.00 | 9.05 |
| NEVPT2(10e,8o)/cc-pVTZ opt. geom. | $MeO_2$ + $MeO_2$ | $MeO_4Me$ | MeO + MeO + $O_2$ |
| NEVPT2(10e,8o)/cc-pVTZ | 14.74 | 0.00 | 28.30 |
| NEVPT2(22e,14o)/cc-pVTZ opt. geom. | $MeO_2$ + $MeO_2$ | $MeO_4Me$ | MeO + MeO + $O_2$ |
| NEVPT2(22e,14o)/cc-pVTZ | 19.68 | 0.00 | 34.35[a] |

[a] Optimized geometry obtained only with very loose convergence criteria, unstable gradient.

# S6 Structure analysis of various studied stationary points

Tables S7-S13 list key structural parameters for $MeO_2$, $MeO_4Me$, MeO+MeO+$O_2$, $^3$(MeO...MeO), $^2MeO_3$, and $^2$(MeO...$O_2$). It appears that the choice of method has an appreciable effect on the structural parameters. Methods used for calculating these parameters were $\omega$B97X-D3 functional, M06-2X functional with zero-damping dispersion correction, MP2, CCSD(T), CASSCF, canonical CASPT2, and NEVPT2. DFT calculations were done with two basis sets: cc-pVDZ and with the fully augmented aug-cc-pVTZ basis set. For the most part, DFT predicts slightly shorter bond lengths than the other used methods. In addition, M06-2X suggests slightly shorter bond lengths than $\omega$B97X-D3. DFT structures do not appear to be very basis set dependent.

CASSCF calculations show the largest structural variations, and these are most apparent for the $MeO_4Me$, $MeO_2$, and $O_2$. The CASSCF calculations with CAS(6e,6o) and

CAS(22e,14o) show almost equal O-O bond lengths in $MeO_4Me$. The CAS(10e,8o) active space likely over-emphasizes the static correlation in the inner O-O bond (O-$O_{in}$ is shortened and O-$O_{out}$ is lengthened in comparison to other active spaces), and the structure is skewed towards decomposition TS-like structure. The addition of dynamical correlation by perturbation (CASPT2 and NEVPT2) or coupled-cluster expansion appears to change the O-O bond lengths towards being more similar. With the CAS(10e,8o), CASPT2 cannot fully compensate for the imbalance in the active space. Then again, NEVPT2 correction shifts the inner O-O bond lengths to be longer than the outer O-O bond lengths, unlike other methods. For $MeO_2$ structure, only the results with CAS(6e,6o) space deviate noticeably from other methods. Both CASPT2 and NEVPT2 yield comparable structures to CCSD(T) result.

The CASSCF structure for methoxyl radical is largely active space independent. The CAS(6e,6o) and CAS(10e,8o) spaces have identical sets of active orbitals localized on MeO fragments, but also the CAS(22e,14o) space, which adds 2s oxygen orbitals and two p-type orbitals, yields identical structure. CASPT2 and NEVPT2 structures have slight differences.

Molecular oxygen is the most sensitive towards the method of choice. It does not come as a surprise, because it is also the most strongly correlated molecule in the reaction. Given that, the various multiconfigurational approaches and CCSD(T) should produce the most reliable results. CASSCF(6e,6o) underestimates the O-O bond length in comparison to the experimental 1.207 Å bond length for triplet molecular oxygen. Other methods are in closer agreement with the experimental value. DFT predicts slightly shorter bond length, while rest of methods predicts slightly longer bond length than the experimental value.

The CASPT2 optimized structure of $^3$(MeO...MeO) is relatively similar to what is obtained with DFT methods, but the $^2$(MeO...$O_2$) geometry is notably different from the DFT-structure. DFT methods suggest two distinct minima for this system: covalently bound trioxyl radical ($MeO_3$, MeO···OO bond length 1.50 Å) and the vdw-complex $^2$(MeO...$O_2$, MeO···OO distance 3.18 Å), of which the latter is the global minimum structure. The

CASPT2 optimized structure is something in-between these two, with a MeO-OO bond length of 1.87 Å.

**Table S7: Structural parameters of $MeO_4Me$ with various levels of theory.**

| $MeO_4Me$ | r(O-O)$_{out}$ (Å) | r(O-O)$_{in}$ (Å) | a(O-O-O) (°) | d(O-O-O-O) (°) |
|---|---|---|---|---|
| CASSCF(6e,6o)/cc-pVDZ | 1.465 | 1.451 | 105.2 | 78.70 |
| CASSCF(10e,8o)/cc-pVDZ | 1.506 | 1.385 | 106.8 | 81.07 |
| CASSCF(22e,14o)/cc-pVDZ | 1.470 | 1.438 | 107.0 | 78.93 |
| CASPT2(6e,6o)/cc-pVDZ | 1.442 | 1.434 | 107.1 | 77.47 |
| CASPT2(10e,8o)/cc-pVDZ | 1.474 | 1.390 | 107.8 | 79.39 |
| CASPT2(22e,14o)/cc-pVDZ | 1.455 | 1.430 | 107.8 | 77.41 |
| NEVPT2(6e,6o)/cc-pVDZ | 1.437 | 1.440 | 106.5 | 76.64 |
| NEVPT2(10e,8o)/cc-pVDZ | 1.434 | 1.444 | 106.4 | 74.88 |
| NEVPT2(22e,14o)/cc-pVDZ | 1.436 | 1.431 | 107.6 | 76.68 |
| CASPT2(22e,14o)/cc-pVTZ | 1.446 | 1.418 | 108.1 | 78.28 |
| NEVPT2(22e,14o)/cc-pVTZ | 1.429 | 1.416 | 108.0 | 77.96 |
| CCSD(T)/cc-pVDZ | 1.443 | 1.436 | 107.4 | 76.44 |
| $\omega$B97X-D3/cc-pVDZ | 1.404 | 1.400 | 108.1 | 76.27 |
| $\omega$B97X-D3/aug-cc-pVTZ | 1.404 | 1.393 | 108.6 | 78.37 |
| M06-2X/aug-cc-pVTZ | 1.402 | 1.391 | 107.9 | 77.24 |
| MP2/aug-cc-pVTZ | 1.429 | 1.417 | 107.6 | 77.38 |

**Table S8: Structural parameters of $MeO_2$ with various levels of theory.**

| $MeO_2$[a] | r(O-O) (Å) | r(C-O) (Å) | a(C-O-O) (°) |
|---|---|---|---|
| CASSCF(6e,6o)/cc-pVDZ | 1.381 | 1.413 | 109.0 |
| CASSCF(10e,8o)/cc-pVDZ | 1.349 | 1.418 | 109.9 |
| CASSCF(22e,14o)/cc-pVDZ | 1.351 | 1.418 | 109.8 |
| CASSCF(22e,14o)/cc-pVTZ | 1.344 | 1.417 | 110.0 |
| CASPT2(6e,6o)/cc-pVDZ | 1.300 | 1.447 | 110.5 |
| CASPT2(10e,8o)/cc-pVDZ | 1.330 | 1.443 | 109.6 |
| CASPT2(22e,14o)/cc-pVDZ | 1.334 | 1.441 | 109.6 |
| NEVPT2(22e,14o)/cc-pVDZ | 1.335 | 1.440 | 110.1 |
| CASPT2(22e,14o)/cc-pVTZ | 1.334 | 1.441 | 109.6 |
| NEVPT2(22e,14o)/cc-pVTZ | 1.328 | 1.435 | 110.3 |
| NEVPT2(11e,7o)/cc-pVTZ[b] | 1.328 | 1.435 | 110.3 |
| CCSD(T)/cc-pVDZ | 1.330 | 1.448 | 109.6 |
| $\omega$B97X-D3/cc-pVDZ | 1.301 | 1.436 | 110.9 |
| $\omega$B97X-D3/aug-cc-pVTZ | 1.300 | 1.437 | 111.2 |
| M06-2X/aug-cc-pVTZ | 1.297 | 1.435 | 111.1 |
| MP2/aug-cc-pVTZ | 1.296 | 1.448 | 110.5 |

[a] MCSCF structures are from supermolecular calculations, in which the $MeO_2$ molecules are 30 Å apart from each other.

[b] The CAS(22e,14o) active space has been split into CAS(11e,7o) for $MeO_2$.

**Table S9: Structural parameters of MeO with various levels of theory.**

| MeO[a] | r(C-O) (Å) | r(C-H) (Å)[b] | a(H-C-O) (°)[b] |
|---|---|---|---|
| CASSCF(6e,6o)/cc-pVDZ | 1.381 | 1.094 | 106.1 |
| CASSCF(10e,8o)/cc-pVDZ | 1.381 | 1.094 | 106.1 |
| CASSCF(22e,14o)/cc-pVDZ | 1.381 | 1.094 | 106.1 |
| CASSCF(22e,14o)/cc-pVTZ | 1.380 | 1.086 | 106.2 |
| CASPT2(6e,6o)/cc-pVDZ | 1.375 | 1.112 | 104.8 |
| CASPT2(10e,8o)/cc-pVDZ | 1.374 | 1.111 | 104.7 |
| CASPT2(22e,14o)/cc-pVDZ | 1.376 | 1.111 | 104.9 |
| NEVPT2(22e,14o)/cc-pVDZ | - | - | - |
| CASPT2(22e,14o)/cc-pVTZ | 1.371 | 1.098 | 104.8 |
| NEVPT2(22e,14o)/cc-pVTZ | - | - | - |
| NEVPT2(5e,3o)/cc-pVTZ[c] | 1.367 | 1.099 | 104.8 |
| CCSD(T)/cc-pVDZ | 1.381 | 1.117 | 105.2 |
| $\omega$B97X-D3/cc-pVDZ | 1.361 | 1.114 | 105.2 |
| $\omega$B97X-D3/aug-cc-pVTZ | 1.360 | 1.105 | 105.1 |
| M06-2X/aug-cc-pVTZ | 1.367 | 1.103 | 105.3 |
| MP2/aug-cc-pVTZ | 1.377 | 1.099 | 104.9 |

[a] MCSCF structures are from supermolecular calculations, in which the MeO molecules are 30 Å apart from the central $O_2$ molecule.
[b] The Jahn-Teller distorted C-H bond in MeO.
[c] The CAS(22e,14o) active space has been split into CAS(5e,3o) for MeO.

**Table S10: Structural parameters of $^3O_2$ with various levels of theory.**

| $^3O_2$[a] | r(O-O) Å |
|---|---|
| CASSCF(6e,6o)/cc-pVDZ | 1.170 |
| CASSCF(10,8o)/cc-pVDZ | 1.219 |
| CASSCF(22e,14o)/cc-pVDZ | 1.221 |
| CASSCF(22e,14o)/cc-pVTZ | 1.218 |
| CASPT2(6e,6o)/cc-pVDZ | 1.260 |
| CASPT2(10,8o)/cc-pVDZ | 1.217 |
| CASPT2(22e,14o)/cc-pVDZ | 1.218 |
| NEVPT2(22e,14o)/cc-pVDZ | - |
| CASPT2(22e,14o)/cc-pVTZ | 1.216 |
| NEVPT2(22e,14o)/cc-pVTZ | - |
| NEVPT2(12e,8o)/cc-pVTZ[b] | 1.212 |
| CCSD(T)/cc-pVDZ | 1.216 |
| $\omega$B97X-D3/cc-pVDZ | 1.197 |
| $\omega$B97X-D3/aug-cc-pVTZ | 1.195 |
| M06-2X/aug-cc-pVTZ | 1.190 |
| MP2/aug-cc-pVTZ | 1.224 |

[a] MCSCF structures are from supermolecular calculations, in which the MeO molecules are 30 Å apart from the central $O_2$ molecule.
[b] The CAS(22e,14o) active space has been split into CAS(12e,8o) for $O_2$.

**Table S11: Structural parameters of MeO...MeO with various levels of theory.**

| $^3$(MeO...MeO) | r(MeO-OMe) Å |
|---|---|
| $\omega$B97X-D3/aug-cc-pVTZ | 3.460 |
| M06-2X/aug-cc-pVTZ | 3.410 |
| CASPT2(22e,14o)/cc-pVTZ | 3.767 |

**Table S12: Structural parameters of $MeO_3$ with various levels of theory.**

| $^2(MeO_3)$ | r(MeO-OO) Å | r(MeOO-O) Å |
|---|---|---|
| $\omega$B97X-D3/aug-cc-pVTZ | 1.503 | 1.237 |
| M06-2X/aug-cc-pVTZ | 1.475 | 1.242 |

**Table S13: Structural parameters of MeO...$O_2$ with various levels of theory.**

| $^2$(MeO...$O_2$) | r(MeO-OO) Å | r(MeOO-O) Å |
|---|---|---|
| $\omega$B97X-D3/aug-cc-pVTZ | 3.176 | 1.195 |
| M06-2X/aug-cc-pVTZ | 2.772 | 1.190 |
| CASPT2(22e,14o)/cc-pVTZ | 1.868 | 1.218 |

# S7 CC//DFT Benchmark

**Table S14: CC//DFT relative total energies of $MeO_2$ + $MeO_2$, $MeO_4Me$, MeO...MeO+$O_2$, MeO...$O_2$+MeO, and MeO + MeO + $O_2$, in kcal $mol^{-1}$.**

| Method[a] | $MeO_2$+$MeO_2$ | $MeO_4Me$ | MeO...MeO[b] | MeO...$O_2$[b] | MeO+MeO+$O_2$ |
|---|---|---|---|---|---|
| M06-2X | 12.81 | 0.00 | 15.40 | 23.80 | 19.17 |
| CC/cc-pVTZ | 14.60 | 0.00 | 16.74 | 19.54 | 20.41 |
| CC/cc-pVQZ | 14.67 | 0.00 | 18.18 | 20.83 | 21.54 |
| CC/CBS(2/3) | 16.43 | 0.00 | 21.79 | 24.44 | 25.22 |
| CC/CBS(3/4) | 15.07 | 0.00 | 19.67 | 22.25 | 23.00 |
| $\omega$B97X-D3 | 8.25 | 0.00 | 9.84 | 18.47 | 13.19 |
| CC/cc-pVTZ | 14.56 | 0.00 | 16.75 | 19.55 | 20.48 |
| CC/cc-pVQZ | 14.64 | 0.00 | 18.16 | 20.77 | 21.58 |
| CC/CBS(2/3) | 16.39 | 0.00 | 21.80 | 24.39 | 25.28 |
| CC/CBS(3/4) | 15.05 | 0.00 | 19.63 | 22.20 | 23.01 |
| W2X | 15.22 | 0.00 | 19.78 | 24.26 | 23.09 |
| W3X-L | 14.42 | 0.00 | 19.30 | 18.80 | 22.87 |

[a] DFT geometry optimizations done with aug-cc-pVTZ basis set, CC: CCSD(T) level single-point energy calculations on DFT optimized geometries, W2X and W3X-L single-point energy corrections calculated on $\omega$B97X-D3 optimized geometries.
[b] MeO...MeO + $^3O_2$ and MeO...$O_2$ + $^2$MeO.

# S8 DFT geometries

## S8.1 $\omega$B97X-D3/aug-cc-pVTZ

**$O_2$**
**0 3**

| | | | |
|---|---|---|---|
| O | 5.441303000000 | 9.775028000000 | 10.189871000000 |
| O | 4.294822000000 | 9.506652000000 | 10.392299000000 |

**MeO**
**0 2**

| | | | |
|---|---|---|---|
| H | 4.745668000000 | 11.217891000000 | 7.531368000000 |
| C | 4.791086000000 | 11.398244000000 | 8.612230000000 |
| H | 4.682750000000 | 12.463824000000 | 8.848386000000 |
| H | 3.917493000000 | 10.887590000000 | 9.055180000000 |
| O | 5.873308000000 | 10.826751000000 | 9.204997000000 |

**$MeO_2$**

**0 2**

| | | | |
|---|---|---|---|
| O | -0.382455000000 | 1.405453000000 | 1.728508000000 |
| O | -0.647883000000 | 1.191100000000 | 0.473919000000 |
| C | 0.001183000000 | -0.000542000000 | 0.001934000000 |
| H | -0.286990000000 | -0.100206000000 | -1.041345000000 |
| H | -0.345491000000 | -0.850715000000 | 0.585786000000 |
| H | 1.078283000000 | 0.118974000000 | 0.099228000000 |

**$MeO_3$**

**0 2**

| | | | |
|---|---|---|---|
| H | 5.066699000000 | 11.895144000000 | 7.753938000000 |
| C | 4.702975000000 | 11.323941000000 | 8.607119000000 |
| H | 4.332229000000 | 12.006945000000 | 9.373145000000 |
| H | 3.905245000000 | 10.651963000000 | 8.286073000000 |
| O | 5.846999000000 | 10.626325000000 | 9.024323000000 |
| O | 5.533963000000 | 9.769333000000 | 10.218684000000 |
| O | 4.364244000000 | 9.852996000000 | 10.612239000000 |

**MeO...$O_2$**

**0 2**

| | | | |
|---|---|---|---|
| H | 4.623147000000 | 11.413969000000 | 7.322053000000 |
| C | 4.803585000000 | 11.644698000000 | 8.378996000000 |
| H | 4.585847000000 | 12.695111000000 | 8.606881000000 |
| H | 4.083755000000 | 11.039267000000 | 8.959610000000 |
| O | 6.029933000000 | 11.246947000000 | 8.809969000000 |
| O | 5.396977000000 | 9.024710000000 | 10.989610000000 |
| O | 4.223186000000 | 9.011278000000 | 10.767213000000 |

**MeO...$O_2$**

**0 4**

| | | | |
|---|---|---|---|
| H | 4.803753000000 | 11.998439000000 | 7.297269000000 |
| C | 4.794554000000 | 11.785037000000 | 8.372866000000 |
| H | 4.483410000000 | 12.659807000000 | 8.956712000000 |
| H | 4.025657000000 | 11.009280000000 | 8.537109000000 |
| O | 5.956058000000 | 11.235642000000 | 8.818736000000 |
| O | 5.440497000000 | 8.696600000000 | 11.061231000000 |
| O | 4.363300000000 | 9.180485000000 | 10.882210000000 |

**$^3$(MeO...MeO)**

**0 3**

| | | | |
|---|---|---|---|
| H | -0.814621000000 | 0.876757000000 | -15.497229000000 |
| C | -0.432025000000 | 0.976885000000 | -14.465429000000 |
| H | -0.450666000000 | 2.038482000000 | -14.197491000000 |
| H | -1.124093000000 | 0.383806000000 | -13.852983000000 |
| O | 0.819359000000 | 0.443309000000 | -14.484964000000 |
| O | -1.695935000000 | -1.918514000000 | -14.749336000000 |
| C | -0.539249000000 | -2.617980000000 | -14.622935000000 |
| H | -0.301563000000 | -3.220554000000 | -15.508082000000 |
| H | -0.493420000000 | -3.211438000000 | -13.701794000000 |
| H | 0.252004000000 | -1.844750000000 | -14.545976000000 |

**$MeO_4Me$**
**0 1**

| | | | |
|---|---|---|---|
| H | 0.022391000000 | 3.133694000000 | -0.870681000000 |
| C | -0.066048000000 | 2.426373000000 | -0.048307000000 |
| H | 0.397647000000 | 2.836632000000 | 0.849705000000 |
| H | -1.117961000000 | 2.202062000000 | 0.131186000000 |
| O | 0.629395000000 | 1.272953000000 | -0.493385000000 |
| O | 0.618593000000 | 0.320307000000 | 0.538280000000 |
| O | -0.618512000000 | -0.320315000000 | 0.538394000000 |
| O | -0.629501000000 | -1.272965000000 | -0.493264000000 |
| C | 0.066039000000 | -2.426371000000 | -0.048300000000 |
| H | -0.022473000000 | -3.133679000000 | -0.870677000000 |
| H | -0.397535000000 | -2.836665000000 | 0.849759000000 |
| H | 1.117965000000 | -2.202025000000 | 0.131072000000 |

## S8.2 M06-2X/aug-cc-pVTZ

**$O_2$**
**0 3**

| | | | |
|---|---|---|---|
| O | 5.438946000000 | 9.774476000000 | 10.190287000000 |
| O | 4.297179000000 | 9.507204000000 | 10.391883000000 |

**MeO**
**0 2**

| | | | |
|---|---|---|---|
| C | 0.036505000000 | 0.000290000000 | -0.003987000000 |
| H | -1.010074000000 | -0.008922000000 | 0.342757000000 |
| H | 0.513237000000 | -0.901001000000 | 0.393819000000 |
| H | 0.497240000000 | 0.909815000000 | 0.393949000000 |
| O | -0.036907000000 | -0.000181000000 | -1.369478000000 |

**$MeO_2$**

**0 2**

| | | | |
|---|---|---|---|
| O | -0.380853000000 | 1.399920000000 | 1.726123000000 |
| O | -0.646408000000 | 1.190054000000 | 0.473643000000 |
| C | 0.000879000000 | -0.000173000000 | 0.000606000000 |
| H | -0.286671000000 | -0.101657000000 | -1.041721000000 |
| H | -0.346864000000 | -0.846148000000 | 0.588357000000 |
| H | 1.076563000000 | 0.122069000000 | 0.101023000000 |

**$MeO_3$**

**0 2**

| | | | |
|---|---|---|---|
| H | 5.070143000000 | 11.884164000000 | 7.751903000000 |
| C | 4.699799000000 | 11.324591000000 | 8.608941000000 |
| H | 4.337690000000 | 12.010165000000 | 9.374351000000 |
| H | 3.901084000000 | 10.651858000000 | 8.298504000000 |
| O | 5.842449000000 | 10.620852000000 | 9.034455000000 |
| O | 5.540535000000 | 9.785190000000 | 10.212360000000 |
| O | 4.360651000000 | 9.849827000000 | 10.595009000000 |

**MeO...$O_2$**

**0 2**

| | | | |
|---|---|---|---|
| H | 4.654788000000 | 11.395486000000 | 7.367772000000 |
| C | 4.801325000000 | 11.574042000000 | 8.437827000000 |
| H | 4.584661000000 | 12.612074000000 | 8.707693000000 |
| H | 4.069224000000 | 10.940330000000 | 8.966664000000 |
| O | 6.029908000000 | 11.159136000000 | 8.871411000000 |
| O | 5.396376000000 | 9.237162000000 | 10.765631000000 |
| O | 4.210147000000 | 9.157748000000 | 10.717334000000 |

**MeO...$O_2$**

**0 4**

| | | | |
|---|---|---|---|
| H | 4.764437000000 | 11.472513000000 | 7.286856000000 |
| C | 4.759565000000 | 11.641420000000 | 8.368126000000 |
| H | 4.660907000000 | 12.703849000000 | 8.610678000000 |
| H | 3.868463000000 | 11.132197000000 | 8.769744000000 |
| O | 5.825075000000 | 11.054370000000 | 8.995113000000 |
| O | 5.491765000000 | 8.893588000000 | 11.051539000000 |
| O | 4.376217000000 | 9.178042000000 | 10.752276000000 |

**$^3$(MeO...MeO)**

**0 3**

| | | | |
|---|---|---|---|
| H | -0.441614000000 | 1.523931000000 | -15.473705000000 |
| C | -0.417033000000 | 0.897124000000 | -14.577260000000 |
| H | -0.613516000000 | 1.478407000000 | -13.670398000000 |
| H | -1.236283000000 | 0.158446000000 | -14.660648000000 |
| O | 0.724038000000 | 0.151937000000 | -14.478748000000 |
| O | -1.842833000000 | -2.092860000000 | -14.522206000000 |
| C | -0.574388000000 | -2.586640000000 | -14.650407000000 |
| H | -0.540924000000 | -3.673739000000 | -14.756756000000 |
| H | -0.043994000000 | -2.301888000000 | -13.725888000000 |
| H | -0.025984000000 | -2.078900000000 | -15.453507000000 |

**$MeO_4Me$**

**0 1**

| | | | |
|---|---|---|---|
| H | 0.022391000000 | 3.133694000000 | -0.870681000000 |
| C | -0.066048000000 | 2.426373000000 | -0.048307000000 |
| H | 0.397647000000 | 2.836632000000 | 0.849705000000 |
| H | -1.117961000000 | 2.202062000000 | 0.131186000000 |
| O | 0.629395000000 | 1.272953000000 | -0.493385000000 |
| O | 0.618593000000 | 0.320307000000 | 0.538280000000 |
| O | -0.618512000000 | -0.320315000000 | 0.538394000000 |
| O | -0.629501000000 | -1.272965000000 | -0.493264000000 |
| C | 0.066039000000 | -2.426371000000 | -0.048300000000 |
| H | -0.022473000000 | -3.133679000000 | -0.870677000000 |
| H | -0.397535000000 | -2.836665000000 | 0.849759000000 |
| H | 1.117965000000 | -2.202025000000 | 0.131072000000 |

# S9 CASSCF geometries

## S9.1 CAS(6e,6o)/cc-pVDZ

**$MeO_2 + MeO_2$**

**0 1**

| | | | |
|---|---|---|---|
| H | 10.225708000000 | 3.644601000000 | -9.454845000000 |
| C | 10.753685000000 | 3.896143000000 | -10.373806000000 |
| O | 9.940703000000 | 4.690655000000 | -11.212420000000 |
| O | 8.796532000000 | 3.985502000000 | -11.528881000000 |
| O | -8.768321000000 | -3.603277000000 | 11.577100000000 |
| O | -9.643306000000 | -2.535645000000 | 11.619042000000 |
| C | -10.719981000000 | -2.787732000000 | 10.740964000000 |
| H | -10.365375000000 | -2.888971000000 | 9.715828000000 |
| H | 11.619675000000 | 4.514857000000 | -10.149604000000 |
| H | -11.365494000000 | -1.916426000000 | 10.826780000000 |
| H | -11.261004000000 | -3.684128000000 | 11.041687000000 |
| H | 11.064231000000 | 2.988199000000 | -10.889536000000 |

**$MeO_4Me$**

**0 1**

| | | | |
|---|---|---|---|
| H | 2.084200000000 | -0.223834000000 | 1.410116000000 |
| C | 2.456664000000 | 0.393937000000 | 0.593947000000 |
| O | 1.422182000000 | 1.158131000000 | 0.025898000000 |
| O | 0.457726000000 | 0.217054000000 | -0.549808000000 |
| O | -0.430677000000 | -0.133388000000 | 0.542847000000 |
| O | -1.354145000000 | 0.997763000000 | 0.665082000000 |
| C | -2.408246000000 | 0.770047000000 | -0.237050000000 |
| H | -2.048219000000 | 0.758901000000 | -1.265037000000 |
| H | 3.157993000000 | 1.130740000000 | 0.982417000000 |
| H | -3.078261000000 | 1.616104000000 | -0.092414000000 |
| H | -2.929166000000 | -0.158696000000 | -0.004724000000 |
| H | 2.947003000000 | -0.222982000000 | -0.158964000000 |

**$MeO + MeO + O_2$**

**0 1**

| | | | |
|---|---|---|---|
| H | 27.910816000000 | -1.039673000000 | 2.137763000000 |
| C | 28.278650000000 | -0.456535000000 | 1.289537000000 |
| O | 27.274620000000 | 0.293560000000 | 0.709159000000 |
| O | 0.224939000000 | -9.815066000000 | -3.764762000000 |
| O | -0.592811000000 | -10.067587000000 | -2.966991000000 |
| O | -27.210956000000 | 1.641296000000 | -0.092626000000 |
| C | -28.233977000000 | 1.431173000000 | -0.996413000000 |
| H | -27.876962000000 | 1.467134000000 | -2.028928000000 |
| H | 29.017584000000 | 0.260393000000 | 1.659844000000 |
| H | -28.942493000000 | 2.251769000000 | -0.848883000000 |
| H | -28.755504000000 | 0.489547000000 | -0.806087000000 |
| H | 28.768635000000 | -1.109125000000 | 0.562271000000 |

## S9.2 CAS(10e,8o)/cc-pVDZ

**$MeO_2 + MeO_2$**

**0 1**

| | | | |
|---|---|---|---|
| H | 10.237357000000 | 3.504473000000 | -9.671369000000 |
| C | 10.644702000000 | 4.014503000000 | -10.541423000000 |
| O | 9.611857000000 | 4.709345000000 | -11.220416000000 |
| O | 8.677097000000 | 3.836314000000 | -11.648841000000 |
| O | -8.647667000000 | -3.719826000000 | 11.648312000000 |
| O | -9.545926000000 | -2.733923000000 | 11.849892000000 |
| C | -10.596503000000 | -2.838543000000 | 10.903348000000 |
| H | -10.199129000000 | -2.732033000000 | 9.896479000000 |
| H | 11.356359000000 | 4.777343000000 | -10.235567000000 |
| H | -11.276280000000 | -2.021384000000 | 11.131566000000 |
| H | -11.101346000000 | -3.795352000000 | 11.015419000000 |
| H | 11.116532000000 | 3.302856000000 | -11.215092000000 |

**$MeO_4Me$**

**0 1**

| | | | |
|---|---|---|---|
| H | 2.098215000000 | -0.213892000000 | 1.419779000000 |
| C | 2.478025000000 | 0.387844000000 | 0.594287000000 |
| O | 1.463290000000 | 1.178561000000 | 0.032278000000 |
| O | 0.433992000000 | 0.229533000000 | -0.521172000000 |
| O | -0.406176000000 | -0.106977000000 | 0.527320000000 |
| O | -1.394420000000 | 1.019350000000 | 0.672843000000 |
| C | -2.429804000000 | 0.766167000000 | -0.240751000000 |
| H | -2.061759000000 | 0.773161000000 | -1.266554000000 |
| H | 3.205405000000 | 1.104778000000 | 0.973181000000 |
| H | -3.126681000000 | 1.591541000000 | -0.100189000000 |
| H | -2.929078000000 | -0.177985000000 | -0.021230000000 |
| H | 2.946046000000 | -0.248306000000 | -0.157483000000 |

**$MeO + MeO + O_2$**

**0 1**

| | | | |
|---|---|---|---|
| H | 27.796872000000 | -0.966809000000 | 2.151757000000 |
| C | 28.167347000000 | -0.372286000000 | 1.312611000000 |
| O | 27.168322000000 | 0.398763000000 | 0.751318000000 |
| O | 0.192082000000 | -9.922142000000 | -3.863161000000 |
| O | -0.565006000000 | -10.213513000000 | -2.953574000000 |
| O | -27.100488000000 | 1.746822000000 | -0.063820000000 |
| C | -28.119482000000 | 1.508297000000 | -0.965137000000 |
| H | -27.760346000000 | 1.529619000000 | -1.997341000000 |
| H | 28.915170000000 | 0.330155000000 | 1.692731000000 |
| H | -28.837287000000 | 2.323607000000 | -0.834072000000 |
| H | -28.630331000000 | 0.564182000000 | -0.758857000000 |
| H | 28.646778000000 | -1.016824000000 | 0.571269000000 |

## S9.3 CAS(22e,14o)/cc-pVDZ

**$MeO_2 + MeO_2$**

**0 1**

| | | | |
|---|---|---|---|
| H | 12.113347000000 | 9.987363000000 | -1.238385000000 |
| C | 11.951428000000 | 9.730770000000 | -0.194404000000 |
| H | 12.304451000000 | 10.530091000000 | 0.453031000000 |
| H | 10.898709000000 | 9.527680000000 | -0.011604000000 |
| O | 12.714304000000 | 8.556506000000 | 0.030807000000 |
| O | 12.580261000000 | 8.163152000000 | 1.316610000000 |
| O | -12.587026000000 | -8.164755000000 | 1.232516000000 |
| O | -12.714260000000 | -8.556550000000 | -0.054454000000 |
| C | -11.950051000000 | -9.730439000000 | -0.277095000000 |
| H | -12.107486000000 | -9.986511000000 | -1.321888000000 |
| H | -12.305509000000 | -10.530218000000 | 0.368428000000 |
| H | -10.898169000000 | -9.527087000000 | -0.089779000000 |

**$MeO_4Me$**

**0 1**

| | | | |
|---|---|---|---|
| H | -0.048858000000 | 3.182422000000 | -0.870601000000 |
| C | -0.061675000000 | 2.472367000000 | -0.045097000000 |
| H | 0.476509000000 | 2.886142000000 | 0.807656000000 |
| H | -1.092139000000 | 2.246897000000 | 0.226514000000 |
| O | 0.588512000000 | 1.331607000000 | -0.548233000000 |
| O | 0.633157000000 | 0.341095000000 | 0.536652000000 |
| O | -0.633157000000 | -0.341095000000 | 0.536652000000 |
| O | -0.588512000000 | -1.331607000000 | -0.548233000000 |
| C | 0.061675000000 | -2.472367000000 | -0.045097000000 |
| H | 0.048858000000 | -3.182422000000 | -0.870601000000 |
| H | -0.476509000000 | -2.886142000000 | 0.807656000000 |
| H | 1.092139000000 | -2.246897000000 | 0.226514000000 |

**$MeO + MeO + O_2$**

**0 1**

| | | | |
|---|---|---|---|
| H | 3.933522000000 | 28.452993000000 | -0.809247000000 |
| C | 3.869802000000 | 27.718313000000 | -0.000953000000 |
| H | 4.323387000000 | 28.151793000000 | 0.894159000000 |
| H | 2.814781000000 | 27.494270000000 | 0.176872000000 |
| O | 4.562035000000 | 26.606293000000 | -0.438934000000 |
| O | 0.482233000000 | 0.306240000000 | 11.701924000000 |
| O | -0.575899000000 | -0.302174000000 | 11.700038000000 |
| O | -4.565471000000 | -26.608479000000 | -0.457251000000 |
| C | -3.868437000000 | -27.717546000000 | -0.019399000000 |
| H | -3.930257000000 | -28.452962000000 | -0.827171000000 |
| H | -4.319241000000 | -28.152223000000 | 0.876536000000 |
| H | -2.814078000000 | -27.489294000000 | 0.156999000000 |

## S9.4 CAS(6e,6o)/cc-pVTZ

**$MeO_2 + MeO_2$**

**0 1**

| | | | |
|---|---|---|---|
| H | 10.238486000000 | 3.656206000000 | -9.450011000000 |
| C | 10.750212000000 | 3.902685000000 | -10.369619000000 |
| O | 9.919443000000 | 4.674402000000 | -11.208265000000 |
| O | 8.803533000000 | 3.951333000000 | -11.531476000000 |
| O | -8.779163000000 | -3.621978000000 | 11.566009000000 |
| O | -9.642405000000 | -2.561914000000 | 11.627185000000 |
| C | -10.715411000000 | -2.780491000000 | 10.738597000000 |
| H | -10.357085000000 | -2.863173000000 | 9.722228000000 |
| H | 11.605156000000 | 4.527964000000 | -10.159380000000 |
| H | -11.349751000000 | -1.912010000000 | 10.836032000000 |
| H | -11.263460000000 | -3.670181000000 | 11.014958000000 |
| H | 11.067497000000 | 3.000936000000 | -10.873950000000 |

**$MeO_4Me$**

**0 1**

| | | | |
|---|---|---|---|
| H | 2.097431000000 | -0.224065000000 | 1.407693000000 |
| C | 2.463363000000 | 0.387515000000 | 0.594743000000 |
| O | 1.430653000000 | 1.159292000000 | 0.039378000000 |
| O | 0.459929000000 | 0.243207000000 | -0.528104000000 |
| O | -0.431690000000 | -0.099373000000 | 0.541410000000 |
| O | -1.362406000000 | 1.007146000000 | 0.655148000000 |
| C | -2.415159000000 | 0.765658000000 | -0.241488000000 |
| H | -2.061481000000 | 0.757726000000 | -1.263091000000 |
| H | 3.174520000000 | 1.107828000000 | 0.973864000000 |
| H | -3.095697000000 | 1.593285000000 | -0.099259000000 |
| H | -2.915795000000 | -0.164606000000 | -0.009066000000 |
| H | 2.933386000000 | -0.229834000000 | -0.158919000000 |

**$MeO + MeO + O_2$**

**0 1**

| | | | |
|---|---|---|---|
| H | 27.833822000000 | -0.739755000000 | 1.551273000000 |
| C | 28.230820000000 | -0.251516000000 | 0.667992000000 |
| O | 27.257682000000 | 0.470670000000 | 0.007048000000 |
| O | 0.208023000000 | -9.958569000000 | -3.821050000000 |
| O | -0.579157000000 | -10.220429000000 | -2.999648000000 |
| O | -27.176165000000 | 1.404146000000 | 0.546594000000 |
| C | -28.187833000000 | 1.213972000000 | -0.373056000000 |
| H | -27.821269000000 | 1.292575000000 | -1.390712000000 |
| H | 28.983059000000 | 0.466463000000 | 0.981649000000 |
| H | -28.906599000000 | 2.010703000000 | -0.204727000000 |
| H | -28.688656000000 | 0.263798000000 | -0.222932000000 |
| H | 28.701705000000 | -0.976747000000 | 0.013394000000 |

## S9.5 CAS(10e,8o)/cc-pVTZ

**$MeO_2 + MeO_2$**

**0 1**

| | | | |
|---|---|---|---|
| H | 10.236723000000 | 3.506155000000 | -9.676618000000 |
| C | 10.642121000000 | 4.014422000000 | -10.538058000000 |
| O | 9.609291000000 | 4.702988000000 | -11.220091000000 |
| O | 8.680087000000 | 3.833749000000 | -11.647448000000 |
| O | -8.650720000000 | -3.720877000000 | 11.645701000000 |
| O | -9.543580000000 | -2.738833000000 | 11.845756000000 |
| C | -10.593888000000 | -2.836632000000 | 10.900600000000 |
| H | -10.198588000000 | -2.733150000000 | 9.901709000000 |
| H | 11.345850000000 | 4.773006000000 | -10.231037000000 |
| H | -11.266258000000 | -2.022866000000 | 11.125658000000 |
| H | -11.098942000000 | -3.783962000000 | 11.010968000000 |
| H | 11.114959000000 | 3.309777000000 | -11.204831000000 |

**$MeO_4Me$**

| | | | |
|---|---|---|---|
| **0 1** | | | |
| H | 2.088302000000 | -0.192354000000 | 1.424336000000 |
| C | 2.456988000000 | 0.395530000000 | 0.595288000000 |
| O | 1.426211000000 | 1.148591000000 | 0.011986000000 |
| O | 0.456519000000 | 0.212480000000 | -0.534967000000 |
| O | -0.429458000000 | -0.128092000000 | 0.528252000000 |
| O | -1.358675000000 | 0.981908000000 | 0.670435000000 |
| C | -2.408497000000 | 0.772138000000 | -0.237151000000 |
| H | -2.051033000000 | 0.792696000000 | -1.257276000000 |
| H | 3.165295000000 | 1.127600000000 | 0.956903000000 |
| H | -3.085978000000 | 1.598432000000 | -0.073905000000 |
| H | -2.914934000000 | -0.162020000000 | -0.034510000000 |
| H | 2.932312000000 | -0.243134000000 | -0.137083000000 |

**$MeO + MeO + O_2$**

| | | | |
|---|---|---|---|
| **0 1** | | | |
| H | 27.796696000000 | -0.961819000000 | 2.145412000000 |
| C | 28.166593000000 | -0.372392000000 | 1.313626000000 |
| O | 27.167906000000 | 0.396610000000 | 0.751850000000 |
| O | 0.191268000000 | -9.923329000000 | -3.862469000000 |
| O | -0.564226000000 | -10.214067000000 | -2.954852000000 |
| O | -27.100142000000 | 1.745415000000 | -0.065551000000 |
| C | -28.118720000000 | 1.508799000000 | -0.966019000000 |
| H | -27.760069000000 | 1.529746000000 | -1.989272000000 |
| H | 28.909742000000 | 0.324060000000 | 1.691308000000 |
| H | -28.832096000000 | 2.317704000000 | -0.836690000000 |
| H | -28.623746000000 | 0.571277000000 | -0.760819000000 |
| H | 28.640426000000 | -1.012132000000 | 0.577201000000 |

## S9.6 CAS(22e,14o)/cc-pVTZ

**$MeO_2 + MeO_2$**

**0 1**

| | | | |
|---|---|---|---|
| H | 12.104360000000 | 9.984304000000 | -1.230967000000 |
| C | 11.948183000000 | 9.729087000000 | -0.193915000000 |
| H | 12.299488000000 | 10.523292000000 | 0.446679000000 |
| H | 10.904317000000 | 9.527565000000 | -0.008589000000 |
| O | 12.710981000000 | 8.556947000000 | 0.032089000000 |
| O | 12.580706000000 | 8.162490000000 | 1.310645000000 |
| O | -12.587430000000 | -8.164110000000 | 1.226600000000 |
| O | -12.710951000000 | -8.556998000000 | -0.053109000000 |
| C | -11.946812000000 | -9.728751000000 | -0.276570000000 |
| H | -12.098048000000 | -9.983079000000 | -1.314572000000 |
| H | -12.300921000000 | -10.523608000000 | 0.361663000000 |
| H | -10.903875000000 | -9.527138000000 | -0.086170000000 |

**$MeO_4Me$**

**0 1**

| | | | |
|---|---|---|---|
| H | -0.045600000000 | 3.199203000000 | -0.845892000000 |
| C | -0.063683000000 | 2.479520000000 | -0.039537000000 |
| H | 0.473559000000 | 2.871591000000 | 0.813516000000 |
| H | -1.088278000000 | 2.262248000000 | 0.228335000000 |
| O | 0.574898000000 | 1.340914000000 | -0.555601000000 |
| O | 0.622308000000 | 0.344775000000 | 0.506070000000 |
| O | -0.622308000000 | -0.344775000000 | 0.506070000000 |
| O | -0.574898000000 | -1.340914000000 | -0.555601000000 |
| C | 0.063683000000 | -2.479520000000 | -0.039537000000 |
| H | 0.045600000000 | -3.199203000000 | -0.845892000000 |
| H | -0.473559000000 | -2.871591000000 | 0.813516000000 |
| H | 1.088278000000 | -2.262248000000 | 0.228335000000 |

**$MeO + MeO + O_2$**

**0 1**

| | | | |
|---|---|---|---|
| H | 3.931235000000 | 28.447957000000 | -0.803025000000 |
| C | 3.868556000000 | 27.717736000000 | -0.001273000000 |
| H | 4.319262000000 | 28.145839000000 | 0.887372000000 |
| H | 2.822382000000 | 27.493120000000 | 0.175337000000 |
| O | 4.560347000000 | 26.606141000000 | -0.437287000000 |
| O | 0.481068000000 | 0.305600000000 | 11.702691000000 |
| O | -0.574737000000 | -0.301529000000 | 11.700809000000 |
| O | -4.563771000000 | -26.608321000000 | -0.455603000000 |
| C | -3.867177000000 | -27.716965000000 | -0.019720000000 |
| H | -3.927975000000 | -28.447918000000 | -0.820949000000 |
| H | -4.315146000000 | -28.146254000000 | 0.869736000000 |
| H | -2.821669000000 | -27.488184000000 | 0.155484000000 |

## S9.7 CAS(22e,14o)/aug-cc-pVTZ

**$MeO_2 + MeO_2$**

**0 1**

| | | | |
|---|---|---|---|
| H | 12.103100000000 | 9.986415000000 | -1.230088000000 |
| C | 11.947987000000 | 9.731092000000 | -0.193023000000 |
| H | 12.302144000000 | 10.523734000000 | 0.447837000000 |
| H | 10.904538000000 | 9.529259000000 | -0.006111000000 |
| O | 12.710768000000 | 8.557371000000 | 0.029833000000 |
| O | 12.583071000000 | 8.158862000000 | 1.307487000000 |
| O | -12.589769000000 | -8.160484000000 | 1.223449000000 |
| O | -12.710725000000 | -8.557417000000 | -0.055350000000 |
| C | -11.946623000000 | -9.730757000000 | -0.275676000000 |
| H | -12.096638000000 | -9.985067000000 | -1.313739000000 |
| H | -12.303718000000 | -10.524113000000 | 0.362662000000 |
| H | -10.904134000000 | -9.528894000000 | -0.083497000000 |

**$MeO_4Me$**

**0 1**

| | | | |
|---|---|---|---|
| H | -0.046806000000 | 3.205012000000 | -0.842344000000 |
| C | -0.064176000000 | 2.483477000000 | -0.037794000000 |
| H | 0.476003000000 | 2.871815000000 | 0.815069000000 |
| H | -1.088356000000 | 2.265939000000 | 0.231520000000 |
| O | 0.572388000000 | 1.345430000000 | -0.558910000000 |
| O | 0.621979000000 | 0.344955000000 | 0.499350000000 |
| O | -0.621979000000 | -0.344955000000 | 0.499350000000 |
| O | -0.572388000000 | -1.345430000000 | -0.558910000000 |
| C | 0.064176000000 | -2.483477000000 | -0.037794000000 |
| H | 0.046806000000 | -3.205012000000 | -0.842344000000 |
| H | -0.476003000000 | -2.871815000000 | 0.815069000000 |
| H | 1.088356000000 | -2.265939000000 | 0.231520000000 |

**MeO + MeO + $O_2$**

**0 1**

| | | | |
|---|---|---|---|
| H | 3.931170000000 | 28.449035000000 | -0.803311000000 |
| C | 3.867891000000 | 27.719377000000 | -0.001573000000 |
| H | 4.320011000000 | 28.144687000000 | 0.887448000000 |
| H | 2.822563000000 | 27.491877000000 | 0.175051000000 |
| O | 4.560217000000 | 26.606165000000 | -0.436877000000 |
| O | 0.481040000000 | 0.305597000000 | 11.703076000000 |
| O | -0.574715000000 | -0.301524000000 | 11.701195000000 |
| O | -4.563649000000 | -26.608346000000 | -0.455182000000 |
| C | -3.866499000000 | -27.718601000000 | -0.020023000000 |
| H | -3.927914000000 | -28.448997000000 | -0.821234000000 |
| H | -4.315890000000 | -28.145104000000 | 0.869810000000 |
| H | -2.821848000000 | -27.486942000000 | 0.155191000000 |

# S10 CASPT2 geometries

## S10.1 CAS(6e,6o)/cc-pVDZ

**$MeO_2 + MeO_2$**

**0 1**

| | | | |
|---|---|---|---|
| H | 10.247411000000 | 3.646449000000 | -9.438076000000 |
| C | 10.768361000000 | 3.895392000000 | -10.373395000000 |
| O | 9.888845000000 | 4.669056000000 | -11.223142000000 |
| O | 8.822848000000 | 3.985203000000 | -11.516445000000 |
| O | -8.787884000000 | -3.577286000000 | 11.563218000000 |
| O | -9.617762000000 | -2.578774000000 | 11.632968000000 |
| C | -10.734638000000 | -2.788098000000 | 10.742898000000 |
| H | -10.369015000000 | -2.873759000000 | 9.709722000000 |
| H | 11.630099000000 | 4.547676000000 | -10.180843000000 |
| H | -11.374738000000 | -1.903244000000 | 10.860742000000 |
| H | -11.271814000000 | -3.701506000000 | 11.037852000000 |
| H | 11.075341000000 | 2.982670000000 | -10.903191000000 |

**$MeO_4Me$**

**0 1**

| | | | |
|---|---|---|---|
| H | 2.016092000000 | -0.134591000000 | 1.460420000000 |
| C | 2.417906000000 | 0.402420000000 | 0.585924000000 |
| O | 1.400063000000 | 1.152812000000 | -0.067485000000 |
| O | 0.433313000000 | 0.202734000000 | -0.560197000000 |
| O | -0.408160000000 | -0.152859000000 | 0.544827000000 |
| O | -1.335138000000 | 0.935016000000 | 0.738185000000 |
| C | -2.368999000000 | 0.770896000000 | -0.226467000000 |
| H | -1.973931000000 | 0.859119000000 | -1.251665000000 |
| H | 3.141518000000 | 1.161814000000 | 0.918777000000 |
| H | -3.061230000000 | 1.602191000000 | -0.024685000000 |
| H | -2.888527000000 | -0.193089000000 | -0.097070000000 |
| H | 2.904146000000 | -0.302684000000 | -0.108257000000 |

**$MeO + MeO + O_2$**

**0 1**

| | | | |
|---|---|---|---|
| H | 27.839628000000 | -0.742146000000 | 1.579400000000 |
| C | 28.213712000000 | -0.248535000000 | 0.661997000000 |
| O | 27.253769000000 | 0.490423000000 | 0.010538000000 |
| O | 0.234077000000 | -9.995585000000 | -3.873696000000 |
| O | -0.607017000000 | -10.280835000000 | -2.979816000000 |
| O | -27.171138000000 | 1.422127000000 | 0.555091000000 |
| C | -28.170604000000 | 1.212338000000 | -0.366502000000 |
| H | -27.827311000000 | 1.306437000000 | -1.414635000000 |
| H | 28.976674000000 | 0.494519000000 | 0.981415000000 |
| H | -28.899307000000 | 2.033309000000 | -0.188329000000 |
| H | -28.714115000000 | 0.260001000000 | -0.214773000000 |
| H | 28.727063000000 | -0.976741000000 | 0.005134000000 |

## S10.2 CAS(10e,8o)/cc-pVDZ

**$MeO_2 + MeO_2$**

**0 1**

| | | | |
|---|---|---|---|
| H | 10.116903000000 | 3.555054000000 | -9.612136000000 |
| C | 10.601345000000 | 4.029096000000 | -10.477341000000 |
| O | 9.593336000000 | 4.653206000000 | -11.300434000000 |
| O | 8.728889000000 | 3.734667000000 | -11.722417000000 |
| O | -8.649401000000 | -3.822254000000 | 11.534582000000 |
| O | -9.437402000000 | -2.780502000000 | 11.780445000000 |
| C | -10.568014000000 | -2.801967000000 | 10.889198000000 |
| H | -10.215153000000 | -2.738184000000 | 9.849404000000 |
| H | 11.268887000000 | 4.841519000000 | -10.159777000000 |
| H | -11.167942000000 | -1.921615000000 | 11.154523000000 |
| H | -11.140740000000 | -3.727383000000 | 11.047649000000 |
| H | 11.146346000000 | 3.282138000000 | -11.071387000000 |

**$MeO_4Me$**

**0 1**

| | | | |
|---|---|---|---|
| H | 2.015640000000 | -0.122472000000 | 1.466736000000 |
| C | 2.424610000000 | 0.399506000000 | 0.585785000000 |
| O | 1.425384000000 | 1.168276000000 | -0.070259000000 |
| O | 0.412966000000 | 0.209984000000 | -0.548934000000 |
| O | -0.385208000000 | -0.140856000000 | 0.533673000000 |
| O | -1.357334000000 | 0.946185000000 | 0.747362000000 |
| C | -2.376446000000 | 0.767803000000 | -0.227018000000 |
| H | -1.976231000000 | 0.869267000000 | -1.249380000000 |
| H | 3.165268000000 | 1.144891000000 | 0.913835000000 |
| H | -3.084602000000 | 1.586954000000 | -0.028774000000 |
| H | -2.884023000000 | -0.204304000000 | -0.108220000000 |
| H | 2.897031000000 | -0.321460000000 | -0.102498000000 |

**MeO + MeO + $O_2$**

**0 1**

| | | | |
|---|---|---|---|
| H | 27.860133000000 | -0.945406000000 | 2.197045000000 |
| C | 28.258972000000 | -0.438243000000 | 1.298803000000 |
| O | 27.326155000000 | 0.341397000000 | 0.655572000000 |
| O | 0.195192000000 | -9.641646000000 | -3.792096000000 |
| O | -0.559368000000 | -9.920136000000 | -2.879320000000 |
| O | -27.222633000000 | 1.776612000000 | -0.088533000000 |
| C | -28.214014000000 | 1.441679000000 | -0.979535000000 |
| H | -27.875748000000 | 1.440927000000 | -2.033979000000 |
| H | 29.039581000000 | 0.274985000000 | 1.644820000000 |
| H | -28.968413000000 | 2.251862000000 | -0.880530000000 |
| H | -28.725615000000 | 0.491926000000 | -0.729069000000 |
| H | 28.759389000000 | -1.164085000000 | 0.630546000000 |

## S10.3 CAS(22e,14o)/cc-pVDZ

**$MeO_2 + MeO_2$**

**0 1**

| | | | |
|---|---|---|---|
| H | 12.296170000000 | 9.798758000000 | -1.315277000000 |
| C | 12.006239000000 | 9.710740000000 | -0.259172000000 |
| H | 12.279069000000 | 10.618088000000 | 0.299412000000 |
| H | 10.929399000000 | 9.509923000000 | -0.163606000000 |
| O | 12.750634000000 | 8.588229000000 | 0.254820000000 |
| O | 12.452874000000 | 8.404614000000 | 1.541757000000 |
| O | -12.407829000000 | -8.386388000000 | 1.453927000000 |
| O | -12.709683000000 | -8.559158000000 | 0.166412000000 |
| C | -12.017988000000 | -9.716846000000 | -0.341359000000 |
| H | -12.303914000000 | -9.789563000000 | -1.398952000000 |
| H | -12.341406000000 | -10.608723000000 | 0.213598000000 |
| H | -10.933565000000 | -9.569672000000 | -0.237776000000 |

**$MeO_4Me$**

**0 1**

| | | | |
|---|---|---|---|
| H | -0.001994000000 | 3.148264000000 | -0.884485000000 |
| C | -0.058374000000 | 2.432560000000 | -0.050505000000 |
| H | 0.420582000000 | 2.859075000000 | 0.846640000000 |
| H | -1.113134000000 | 2.188842000000 | 0.155360000000 |
| O | 0.649875000000 | 1.289932000000 | -0.520143000000 |
| O | 0.636226000000 | 0.316907000000 | 0.561782000000 |
| O | -0.644096000000 | -0.319231000000 | 0.559976000000 |
| O | -0.654071000000 | -1.293248000000 | -0.521079000000 |
| C | 0.060236000000 | -2.431847000000 | -0.050852000000 |
| H | 0.005991000000 | -3.149155000000 | -0.883558000000 |
| H | -0.415397000000 | -2.859238000000 | 0.847615000000 |
| H | 1.114155000000 | -2.182861000000 | 0.153032000000 |

**MeO + MeO + $O_2$**

**0 1**

| | | | |
|---|---|---|---|
| H | 3.954151000000 | 28.455326000000 | -0.814918000000 |
| C | 3.872611000000 | 27.717210000000 | 0.011827000000 |
| H | 4.300157000000 | 28.191064000000 | 0.916279000000 |
| H | 2.795804000000 | 27.503424000000 | 0.152358000000 |
| O | 4.589887000000 | 26.624936000000 | -0.419876000000 |
| O | 0.480511000000 | 0.310205000000 | 11.679133000000 |
| O | -0.570554000000 | -0.304641000000 | 11.676136000000 |
| O | -4.584481000000 | -26.623600000000 | -0.445695000000 |
| C | -3.872697000000 | -27.716738000000 | -0.006811000000 |
| H | -3.947863000000 | -28.456532000000 | -0.832689000000 |
| H | -4.308248000000 | -28.188798000000 | 0.894622000000 |
| H | -2.796902000000 | -27.504631000000 | 0.143208000000 |

## S10.4 CAS(6e,6o)/cc-pVTZ

**$MeO_2$ + $MeO_2$**

**0 1**

| | | | |
|---|---|---|---|
| H | 10.230891000000 | 3.634835000000 | -9.445340000000 |
| C | 10.756610000000 | 3.884502000000 | -10.360253000000 |
| O | 9.887022000000 | 4.675078000000 | -11.200274000000 |
| O | 8.822011000000 | 4.008442000000 | -11.501868000000 |
| O | -8.789084000000 | -3.554797000000 | 11.577273000000 |
| O | -9.613664000000 | -2.560363000000 | 11.616388000000 |
| C | -10.723737000000 | -2.788802000000 | 10.723231000000 |
| H | -10.348330000000 | -2.877223000000 | 9.708573000000 |
| H | 11.616171000000 | 4.512882000000 | -10.159399000000 |
| H | -11.369069000000 | -1.922462000000 | 10.825251000000 |
| H | -11.237892000000 | -3.695833000000 | 11.024906000000 |
| H | 11.046125000000 | 2.987522000000 | -10.896184000000 |

**$MeO_4Me$**

**0 1**

| | | | |
|---|---|---|---|
| H | 2.008108000000 | -0.120278000000 | 1.452453000000 |
| C | 2.411663000000 | 0.398817000000 | 0.585640000000 |
| O | 1.401299000000 | 1.153722000000 | -0.068608000000 |
| O | 0.429495000000 | 0.217418000000 | -0.550616000000 |
| O | -0.402991000000 | -0.134924000000 | 0.544467000000 |
| O | -1.335324000000 | 0.935900000000 | 0.738273000000 |
| C | -2.363044000000 | 0.767444000000 | -0.228273000000 |
| H | -1.966996000000 | 0.865609000000 | -1.236857000000 |
| H | 3.137751000000 | 1.140417000000 | 0.908161000000 |
| H | -3.058963000000 | 1.577802000000 | -0.027813000000 |
| H | -2.860549000000 | -0.192360000000 | -0.103424000000 |
| H | 2.876606000000 | -0.305791000000 | -0.101095000000 |

**$MeO + MeO + O_2$**

**0 1**

| | | | |
|---|---|---|---|
| H | 27.852098000000 | -0.750686000000 | 1.562115000000 |
| C | 28.220967000000 | -0.253842000000 | 0.662357000000 |
| O | 27.258139000000 | 0.488129000000 | 0.028225000000 |
| O | 0.233984000000 | -9.982274000000 | -3.867049000000 |
| O | -0.608323000000 | -10.267442000000 | -2.973861000000 |
| O | -27.180448000000 | 1.421947000000 | 0.545759000000 |
| C | -28.177485000000 | 1.208916000000 | -0.370862000000 |
| H | -27.833924000000 | 1.302155000000 | -1.403196000000 |
| H | 28.974211000000 | 0.479092000000 | 0.983944000000 |
| H | -28.898140000000 | 2.020112000000 | -0.196083000000 |
| H | -28.708569000000 | 0.268062000000 | -0.211922000000 |
| H | 28.722921000000 | -0.958857000000 | -0.003603000000 |

## S10.5 CAS(10e,8o)/cc-pVTZ

**$MeO_2 + MeO_2$**

**0 1**

| | | | |
|---|---|---|---|
| H | 10.093431000000 | 3.566343000000 | -9.600775000000 |
| C | 10.582430000000 | 4.025195000000 | -10.454461000000 |
| O | 9.589261000000 | 4.641936000000 | -11.295278000000 |
| O | 8.750191000000 | 3.723622000000 | -11.744869000000 |
| O | -8.643476000000 | -3.825023000000 | 11.503320000000 |
| O | -9.422856000000 | -2.786955000000 | 11.761975000000 |
| C | -10.557909000000 | -2.794568000000 | 10.878455000000 |
| H | -10.207708000000 | -2.728159000000 | 9.853826000000 |
| H | 11.238444000000 | 4.830018000000 | -10.138394000000 |
| H | -11.146127000000 | -1.924005000000 | 11.146415000000 |
| H | -11.122700000000 | -3.707639000000 | 11.034232000000 |
| H | 11.124074000000 | 3.283010000000 | -11.032139000000 |

**$MeO_4Me$**

**0 1**

| | | | |
|---|---|---|---|
| H | 2.008629000000 | -0.109478000000 | 1.457092000000 |
| C | 2.416996000000 | 0.396444000000 | 0.584314000000 |
| O | 1.421735000000 | 1.166289000000 | -0.071340000000 |
| O | 0.410025000000 | 0.221999000000 | -0.537600000000 |
| O | -0.384468000000 | -0.124444000000 | 0.539276000000 |
| O | -1.356839000000 | 0.944645000000 | 0.749642000000 |
| C | -2.368186000000 | 0.765125000000 | -0.229161000000 |
| H | -1.964346000000 | 0.876072000000 | -1.233642000000 |
| H | 3.157847000000 | 1.126015000000 | 0.901587000000 |
| H | -3.078800000000 | 1.564229000000 | -0.033647000000 |
| H | -2.855380000000 | -0.201879000000 | -0.116668000000 |
| H | 2.869841000000 | -0.321243000000 | -0.097545000000 |

**MeO + MeO + $O_2$**

**0 1**

| | | | |
|---|---|---|---|
| H | 26.501601000000 | -0.035132000000 | 2.055949000000 |
| C | 27.321240000000 | -0.222634000000 | 1.358562000000 |
| O | 27.029751000000 | 0.158930000000 | 0.073723000000 |
| O | 0.151041000000 | -10.546712000000 | -4.222355000000 |
| O | -0.485810000000 | -10.720642000000 | -3.203736000000 |
| O | -26.617524000000 | 1.953568000000 | 0.264692000000 |
| C | -27.294226000000 | 1.585803000000 | -0.869272000000 |
| H | -26.741614000000 | 1.807949000000 | -1.782616000000 |
| H | 28.161717000000 | 0.420029000000 | 1.655074000000 |
| H | -28.192995000000 | 2.215859000000 | -0.869260000000 |
| H | -27.626176000000 | 0.546178000000 | -0.847460000000 |
| H | 27.666626000000 | -1.253326000000 | 1.430425000000 |

## S10.6 CAS(22e,14o)/cc-pVTZ

**$MeO_2$ + $MeO_2$**

**0 1**

| | | | |
|---|---|---|---|
| H | 12.422771000000 | 9.921543000000 | -1.284153000000 |
| C | 12.136331000000 | 9.825380000000 | -0.242301000000 |
| H | 12.539713000000 | 10.640878000000 | 0.348304000000 |
| H | 11.058134000000 | 9.771767000000 | -0.137469000000 |
| O | 12.714649000000 | 8.587247000000 | 0.207792000000 |
| O | 12.381088000000 | 8.371886000000 | 1.474865000000 |
| O | -12.481995000000 | -8.415705000000 | 1.412253000000 |
| O | -12.769138000000 | -8.619535000000 | 0.132053000000 |
| C | -12.111571000000 | -9.813797000000 | -0.328049000000 |
| H | -12.392965000000 | -9.920992000000 | -1.370162000000 |
| H | -12.458840000000 | -10.658531000000 | 0.258440000000 |
| H | -11.038273000000 | -9.690160000000 | -0.224795000000 |

**$MeO_4Me$**

**0 1**

| | | | |
|---|---|---|---|
| H | -0.001731000000 | 3.143844000000 | -0.860970000000 |
| C | -0.060875000000 | 2.425764000000 | -0.047333000000 |
| H | 0.406705000000 | 2.834310000000 | 0.846616000000 |
| H | -1.102124000000 | 2.176033000000 | 0.143638000000 |
| O | 0.653642000000 | 1.292157000000 | -0.520422000000 |
| O | 0.635624000000 | 0.314872000000 | 0.545497000000 |
| O | -0.634885000000 | -0.314601000000 | 0.545483000000 |
| O | -0.653012000000 | -1.291744000000 | -0.520586000000 |
| C | 0.060703000000 | -2.425822000000 | -0.047392000000 |
| H | 0.000953000000 | -3.144058000000 | -0.860812000000 |
| H | -0.407150000000 | -2.833900000000 | 0.846616000000 |
| H | 1.102149000000 | -2.176852000000 | 0.143447000000 |

**MeO...MeO (+ $O_2$)**

**0 1**

| | | | |
|---|---|---|---|
| H | -0.435036000000 | 1.339745000000 | -15.647546000000 |
| C | -0.425844000000 | 1.053640000000 | -14.586217000000 |
| H | -0.644936000000 | 1.947732000000 | -13.999516000000 |
| H | -1.190268000000 | 0.284462000000 | -14.461541000000 |
| O | 0.837765000000 | 0.558592000000 | -14.368433000000 |
| O | 1.094262000000 | 0.743363000000 | 15.662013000000 |
| O | -0.116663000000 | 0.854855000000 | 15.650001000000 |
| O | -1.765381000000 | -2.158017000000 | -14.185079000000 |
| C | -0.527631000000 | -2.670188000000 | -14.490491000000 |
| H | -0.622348000000 | -3.030877000000 | -15.524712000000 |
| H | -0.249338000000 | -3.522031000000 | -13.867443000000 |
| H | 0.242809000000 | -1.897053000000 | -14.495242000000 |

**MeO...$O_2$ (+ MeO)**

**0 1**

| | | | |
|---|---|---|---|
| H | 4.715832000000 | 11.240931000000 | 7.515889000000 |
| C | 4.790414000000 | 11.422715000000 | 8.589453000000 |
| H | 4.654183000000 | 12.479709000000 | 8.824565000000 |
| H | 3.949550000000 | 10.880889000000 | 9.062046000000 |
| O | 5.966361000000 | 10.933870000000 | 9.106705000000 |
| O | 5.439220000000 | 9.626184000000 | 10.332082000000 |
| O | 4.230870000000 | 9.491682000000 | 10.403591000000 |
| O | -6.015584000000 | -10.937261000000 | -8.749752000000 |
| C | -4.800374000000 | -11.467870000000 | -9.099180000000 |
| H | -4.990491000000 | -12.010206000000 | -10.035810000000 |
| H | -4.430601000000 | -12.196670000000 | -8.375299000000 |
| H | -4.049293000000 | -10.705255000000 | -9.315177000000 |

**MeO + MeO + $O_2$**

**0 1**

| | | | |
|---|---|---|---|
| H | 3.951789000000 | 28.443913000000 | -0.805120000000 |
| C | 3.869920000000 | 27.715350000000 | 0.012759000000 |
| H | 4.297990000000 | 28.180626000000 | 0.903233000000 |
| H | 2.807753000000 | 27.503484000000 | 0.150716000000 |
| O | 4.582236000000 | 26.625819000000 | -0.417478000000 |
| O | 0.479346000000 | 0.309584000000 | 11.680458000000 |
| O | -0.570298000000 | -0.304702000000 | 11.677608000000 |
| O | -4.579507000000 | -26.625636000000 | -0.441496000000 |
| C | -3.869600000000 | -27.714858000000 | -0.005809000000 |
| H | -3.946154000000 | -28.444954000000 | -0.822936000000 |
| H | -4.302700000000 | -28.178921000000 | 0.882853000000 |
| H | -2.808399000000 | -27.502481000000 | 0.138786000000 |

# References


(1) Roos, B. O.; Fülscher, M.; Malmqvist, P.-Å.; Merchán, M.; Serrano-Andrés, L. Theoretical Studies of the Electronic Spectra of Organic Molecules. *Quantum mechanical electronic structure calculations with chemical accuracy* **1995**, 357–438.
